# A 1-year study on SARS-CoV-2 variant shifts in wastewater using dPCR: comparison with clinical and GISAID data

**DOI:** 10.1128/msystems.00229-25

**Published:** 2025-10-22

**Authors:** Sayed Mohammad R. Mosavi, Patrick Acer, Patrick Andersen, Robbie Barbero, Stephanie Barksdale, Sophia Bellakbira, Dalton Bunde, Ross Dunlap, James Erickson, Daniel Goldfarb, Tara Jones-Roe, Michael Kilroy, Hien Le, Benjamin Lepene, Emily Milich, Ayan Mohamed, Denton Munns, Jared Obermeyer, Anurag Patnaik, Ganit Pricer, Marion T. Reven, Dalaun Richardson, Chamodya Ruhunusiri, Saswata K. Sahoo, Lauren P. Saunders, Olivia Swahn, Kalpita Vengurlekar, David White, Jeremy Davis-Turak, Aaron Stanz, Aouda P. Flores-Baffi, Jean Lozach, Tim Wesselman, Stephen Hilton, Siya Kashwala, Pengbo Liu, Christine L. Moe, Orlando Sablon, Yuke Wang, Marlene Wolfe, Dagmara Antkiewicz, Erica Camarato, Kayley Janssen, Adélaïde Roguet, Shreya Shrestha, Regan Wied, Johannah Gillespie, Jim Huang, Andrew Jones, Sarah Kane, Dolores S. Gonzalez, Modou L. Jarju, Chi-Yu Lin, Mayumi E. Pascual, Rachel Poretsky, Michael Secreto, Ian Bradley, Sydney Gallo, Yinyin Ye, Elizabeth Donahue, Stephanie M. Greenwald, Sarah Owens, Rosemarie Wilton

**Affiliations:** 1 Ceres Nanosciences Inc. 585794, Manassas, Virginia, USA; 2 Rosalind, Inc., San Diego, California, USA; 3 Center for Global Safe Water, Sanitation, and Hygiene at Emory University 1371, Atlanta, Georgia, USA; 4 University of Wisconsin Madison 5228, Madison, Wisconsin, USA; 5 GT Molecular, Fort Collins, Colorado, USA; 6 University of Illinois Chicago 14681, Chicago, Illinois, USA; 7 University at Buffalo-SUNY 12292, Buffalo, New York, USA; 8 Argonne National Laboratory 1291, Lemont, Illinois, USA; University of California, San Diego, La Jolla, California, USA

**Keywords:** wastewater-based epidemiology (WBE), genotyping, SARS-CoV-2, whole-genome sequencing (WGS), digital PCR (dPCR), mutations, variants, Omicron

## Abstract

Wastewater testing can be used to monitor SARS-CoV-2 infections in communities. Data from PCR-based wastewater testing are usually available to public health authorities within 5–7 days after excreta and other body fluids enter the sewer. While PCR-based methods can accurately detect and quantify SARS-CoV-2, sequencing-based methods are usually required to distinguish between variants, delaying the results and adding cost to the process. We developed and assessed a novel, customizable digital PCR (dPCR)-based genotyping method for SARS-CoV-2 variant detection in wastewater, which is more cost-effective, faster, and more accessible than sequencing. This approach was applied to more than 1,400 wastewater samples collected from six states between April 2023 and May 2024, with results displayed on a public dashboard alongside clinical data from the same period. The wastewater dPCR-based method effectively detected emerging variants, mirroring trends observed in clinical settings and in data on the Global Initiative on Sharing All Influenza Data (GISAID) platform; this method also provided early warning signals, as variants like EG.5 and FL were identified in wastewater before clinical detection. A subset of the wastewater samples was analyzed using both dPCR genotyping and sequencing, with good agreement between the two methods. The range of concordance for four different variants was between 62% and 98%. The successful development and implementation of this dPCR-based genotyping for wastewater samples demonstrates its cost-effectiveness and scalability. With the decline in clinical testing, wastewater surveillance becomes increasingly vital for monitoring SARS-CoV-2 variants and supplementing clinical surveillance efforts.

**IMPORTANCE:**

As clinical specimens are collected and analyzed less for SARS-CoV-2, variant detection in wastewater provides a readily accessible and rich source of information on SARS-CoV-2 evolution. The detection of pathogen targets in wastewater samples using PCR assays is a sensitive, cost-effective way to monitor the levels of infectious diseases, like SARS-CoV-2, in a community. Unfortunately, because PCR-based methods are typically not used to distinguish between viral variants, most wastewater testing labs must rely on more expensive, time-consuming, and resource-intensive sequencing methods for these results. Building upon recent developments for variant detection using quantitative PCR, we developed and assessed a novel, customizable digital PCR-based genotyping method for SARS-CoV-2 variant detection in wastewater, which is more rapid, cost-effective, and accessible than sequencing. We characterize the method and offer insights for improvement in future implementations.

## INTRODUCTION

SARS-CoV and SARS-CoV-2 are both coronaviruses, with the former causing a high-fatality outbreak in 2002 and the latter triggering a prolonged global pandemic due to its rapid spread and evolving variants (1–3). Given its widespread impact, effective surveillance strategies are essential to monitor and control future outbreaks (4–6). Because wastewater testing can be used to monitor levels of infectious diseases in a community, it has emerged as an effective tool that provides information for disease prevention and control measures (5–7). PCR-based methods continue to be widely used for detecting and quantifying SARS-CoV-2 in wastewater, and data from these methods are usually available to public health authorities within 5–7 days after excreta enters the sewer (8–11) (https://www.cdc.gov/nwss/how-wws-works.html). As SARS-CoV-2 continues to mutate, giving rise to variants with differing levels of transmissibility and virulence, monitoring the emergence and abundance of these variants has become a pivotal aspect of wastewater-based surveillance (12, 13). Despite being the gold standard for detection and quantification, PCR-based methods are generally not designed to differentiate among the wide array of SARS-CoV-2 variants (14).

Genomic sequencing of isolates from clinical and wastewater samples is the primary method for monitoring SARS-CoV-2 genetic lineages in the United States (15). Laboratory sequencing methods typically are complex, time-consuming, and costly, and many wastewater testing labs do not have access to the instruments or expertise required to sequence samples and interpret the results (15–17).

PCR-based genotyping offers a potential alternative to sequencing for rapid, cost-effective variant detection. By targeting variant-specific mutations, PCR assays can be designed to screen samples for these mutations. In this study, we leveraged the growing trend of using digital PCR (dPCR) systems for wastewater testing and developed a customizable dPCR-based genotyping approach for detecting SARS-CoV-2 variants in wastewater. This method builds on a previously established quantitative PCR (qPCR) approach for clinical sample analysis, shown to provide quantitative results, is effective against potential inhibitors in wastewater samples, and has a low limit of detection (15, 18). Here, we combined this novel approach with a well-established wastewater processing method involving Nanotrap Particle concentration (11) to detect SARS-CoV-2 variants in wastewater samples from six states in the United States. The results were uploaded to a public-facing dashboard (https://tracker.rosalind.bio/) and are presented alongside clinical qPCR-based genotyping and Global Initiative on Sharing All Influenza Data (GISAID) sequence genotyping.

**Fig 1:**
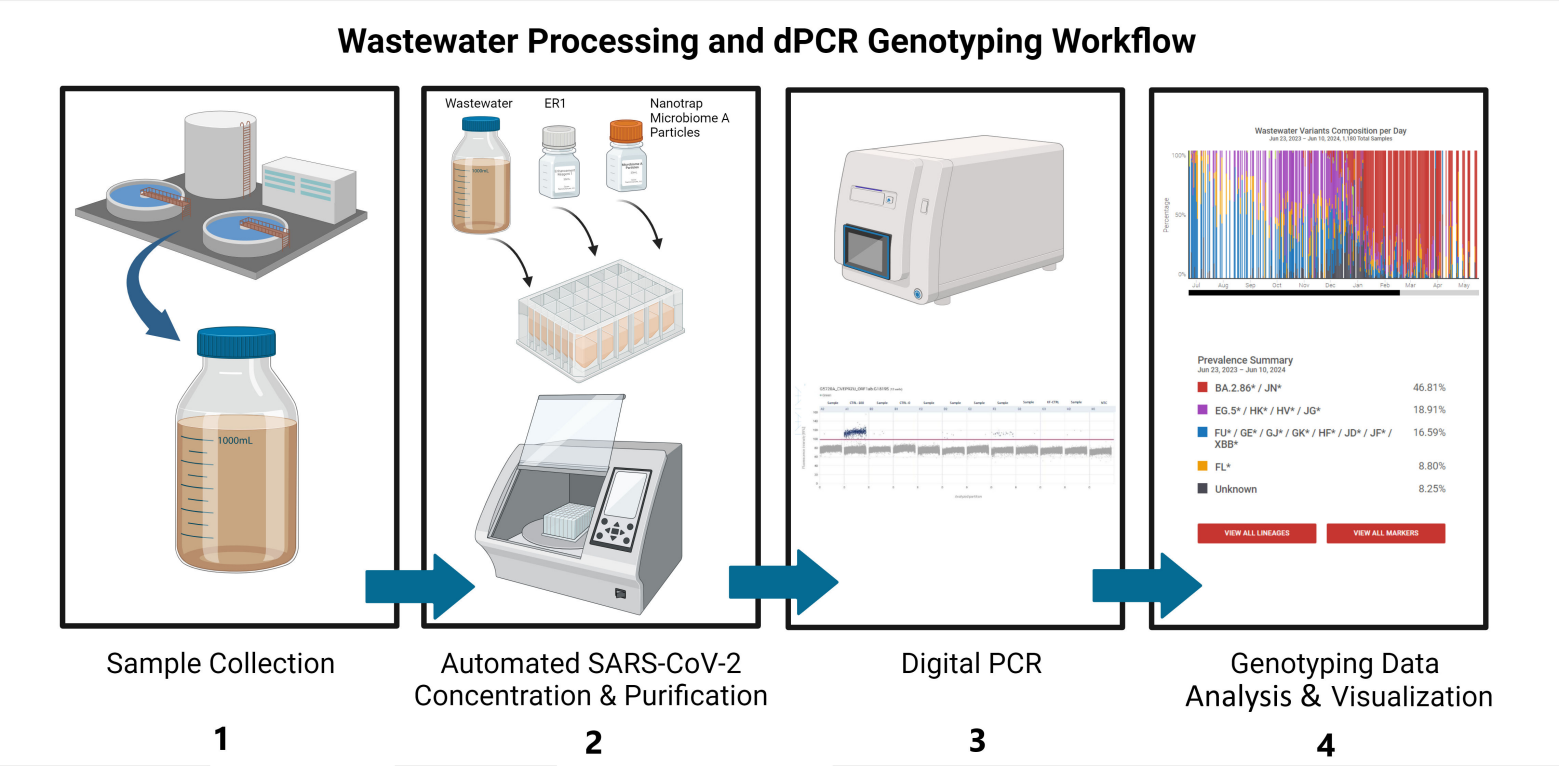
Workflow for wastewater processing and dPCR detection, quantification, and genotyping. (1) Sample collection: untreated wastewater is collected from treatment facilities. (2) Automated SARS-CoV-2 concentration and RNA extraction: using an automated system, viruses are concentrated from the wastewater using Nanotrap Microbiome A Particles, and nucleic acids are purified using a nucleic acid extraction kit. (3) dPCR: samples are analyzed on the QIAGEN QIAcuity Digital PCR system using mutation-specific genotyping assays to detect SARS-CoV-2 variants. (4) Genotyping data analysis and visualization: the composition and prevalence of SARS-CoV-2 variants are analyzed and visualized over time. (Figure was created in BioRender [A. Patnaik, 2025, https://www.biorender.com/u32z193].)

The wastewater processing and dPCR genotyping workflow involves four steps: sample collection, automated viral concentration and nucleic acid extraction, dPCR analysis, and genotyping data analysis and visualization (Fig. 1). Full method details are provided in Materials and Methods and in the Supplemental material. Multiple dPCR-based genotyping panels were developed and deployed to match the changes in SARS-CoV-2 variant prevalence in the United States during the study period (Table 4). We compared the wastewater dPCR-based genotyping data to clinical qPCR-based genotyping data (https://tracker.rosalind.bio/tracker/dashboard). We also compared dPCR variant data to sequence data, both from the sequences deposited in GISAID and wastewater variant genome sequencing data on a subset of the samples. Since January 2020, genomic sequences shared via GISAID have been the primary source of genomic and associated data from SARS-CoV-2 cases (19, 20).

This paper describes the challenges and insights from developing, implementing, and evaluating this dPCR-based approach and highlights its potential as a complementary tool to clinical surveillance for variant detection in communities. This study highlights the utility of wastewater-based genotyping as an effective way to monitor SARS-CoV-2 variants. By integrating dPCR-based genotyping and sequencing methods, we compared the prevalence of variants in wastewater samples with clinical and GISAID data sets, demonstrating a strong correlation and emphasizing the utility of wastewater testing for community-level tracking of variants.

## RESULTS

### Developing the dPCR-based wastewater genotyping method

We began by demonstrating the feasibility of qPCR-based genotyping in wastewater samples using archived nucleic acids from 10 samples in Georgia during the BA.1-to-BA.2 variant transition (February–April 2022). The results revealed a strong correlation between whole-genome sequencing (WGS) and qPCR results, with a *t*-test *P*-value of 0.9269 and 0.1983 and *r*-values of 0.6514 and 0.9922 for BA.1 and BA.2, respectively (Fig. S4).

Next, we applied dPCR-based genotyping to nine archived samples from Georgia collected during the BQ-to-XBB variant transition (January–March 2023). The results indicated no significant differences between the two methods and showed a strong correlation, with *t*-test *P-*values of 0.8342 and 0.8685 and correlation coefficients of 0.8256 and 0.8537 for BQ.1 and XBB, respectively (Fig. S5). Based on these findings, we developed a standard operating procedure for a 2-marker panel to monitor BQ.1 and XBB variants, which was used to test 86 samples in Georgia between 11 April and 19 June 2023. Wastewater genotyping results aligned with clinical data, with XBB being the dominant variant detected (88.23% vs 96.39%; Fig. S7).

In June 2023, we updated the variant panel to reflect the evolving shift in prevalence by adding markers for XBB, EG, FD, and FL. This updated panel was used to evaluate 73 archived samples collected between June and September 2023, confirming XBB as the most prevalent variant in Georgia during this period (72.81% in wastewater samples and 66.47% in clinical samples). Notably, the FL and EG.5 variants were detected 22 and 31 days earlier in wastewater samples than in clinical data (Fig. S9).

Finally, in late 2023, as the JN variant began rising in prevalence, we validated a new assay to detect XBB, EG.5, FL, and JN in wastewater samples, enabling continued monitoring during early 2024 (Supplemental material).

### Wastewater genotyping results correlate with and complement clinical genotyping and GISAID results over a 1-year timeframe in Georgia

**Fig 2:**
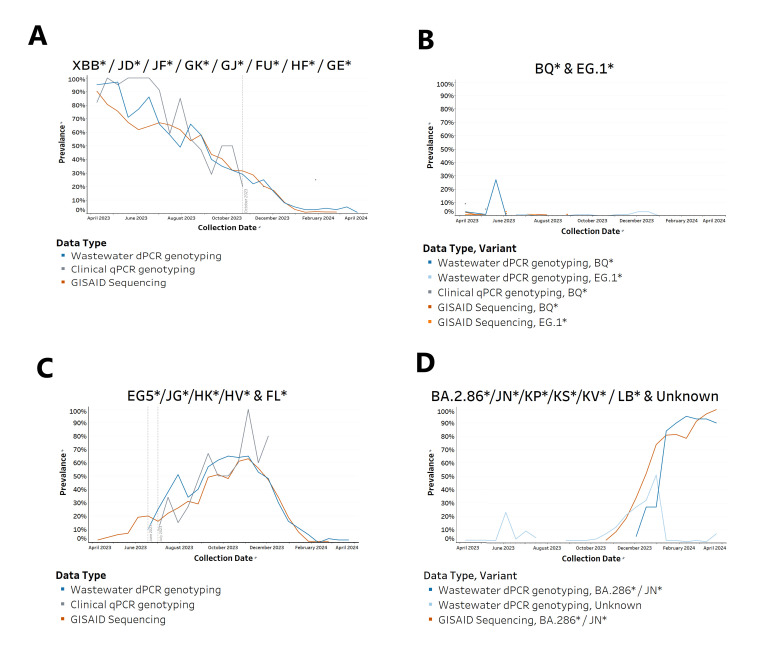
Prevalence of variants across clinical, GISAID, and wastewater data in Georgia (2-week averages, April 2023–April 2024). A total of 528 wastewater samples were processed and analyzed using dPCR-based genotyping panels, alongside 330 clinical qPCR-based genotyping samples and 2,855 clinical sequencing data sets from GISAID. (**A**) The prevalence of XBB* variants detected in clinical samples, GISAID sequences, and wastewater samples. A significant decline in the XBB* variant is observed across all data sets over the study period. In October 2023, the prevalence of the XBB* variant was still at 30% prevalence in both wastewater and GISAID results, but it disappeared from the clinical genotyping results. The vertical dotted line demarcates the last date that the clinical genotyping results reported XBB*. (**B**) The prevalence of BQ* and EG.1* variants detected in wastewater, clinical, and GISAID data sets. A notable spike in the BQ* variant is observed in wastewater samples in May 2023, reaching a prevalence of 27%, which is not reflected in clinical or GISAID data. The EG.1* variant was only detected in the wastewater data set during the fall and winter of 2023, with a prevalence of 1% in September and 3% in December, and showed no signal in the clinical and GISAID data sets. (**C**) The prevalence of EG.5* and FL* variants detected in wastewater, clinical, and GISAID data sets. The data show that EG.5/FL was detected in wastewater samples 22 days earlier than in clinical samples. The first vertical dotted line demarcates the date that EG.5 and FL were first reported in wastewater genotyping samples in Georgia, and the second vertical dotted line demarcates the date that EG.5 and FL were first reported in clinical genotyping samples in Georgia. (**D**) The prevalence of the BA.2.86*/JN* variant and an unknown marker detected in wastewater samples and the GISAID data set. The second peak of the unknown marker in the wastewater data set from October 2023 to January 2024 is associated with the BA.2.86*/JN* variant. Note that because there were very few clinical genotyping results during this time period, they were not included in the figure.

Using state-level data in Georgia between April 2023 and April 2024, we examined variant prevalence reported by three different methods—wastewater genotyping, clinical sample genotyping, and GISAID—for several variants: XBB, BQ, EG.1, EG.5/FL, and JN (Fig. 2). The wastewater genotyping results for XBB tracked a steady decline in variant prevalence from nearly 100% in April 2023 until it almost completely disappeared by January 2024, a result that was mirrored in the GISAID data (Fig. 2A). The clinical genotyping data similarly tracked this decline until October 2023 when it stopped reporting XBB completely.

Because the BQ and EG.1 variants were both present at low levels during the study period in Georgia, we combined these results into a single chart (Fig. 2B). For most of the study period, the wastewater genotyping results were consistent with the reported prevalences of these variants in clinical genotyping and GISAID results. Notably, however, the wastewater testing results showed an increase in the BQ variant to 27% prevalence in May 2023, which was not reported in either of the other surveillance approaches.

While the wastewater genotyping panel 2 was able to distinguish between EG.5 and FL variants, the genotyping panel that was used for clinical samples between July 2023 and October 2023 could not distinguish between these two variants. Thus, to facilitate a comparison between the wastewater genotyping and clinical genotyping in Georgia during this time period, we combined the prevalence of EG.5 and FL into one chart (Fig. 2C). The wastewater genotyping results track closely with the clinical genotyping and GISAID results. Also, the data indicate that EG.5/FL was detected in wastewater samples 22 days earlier than in clinical samples (Fig. 2C).

Fig. 2D illustrates the rapid rise and dominance of the BA.2.86*/JN* variant detected in wastewater samples and the GISAID data sets between October 2023 and January 2024. The “unknown variant” represents SARS-CoV-2 signals that did not match known variant-specific mutations in the genotyping panel, likely indicating emerging variants. The upward trend of the unknown marker in wastewater, which began in October 2023 and peaked at around 50% in January 2024, sharply declined after the transition to the new panel with the JN assay in February 2024.

### Wastewater dPCR-based genotyping shows strong agreement with whole-genome sequencing for variant detection

We compared the performance of the dPCR genotyping method with WGS using 339 data points from the ROSALIND wastewater dashboard (panel 3) using data from Wisconsin and Illinois, where corresponding sequencing data were available. Details of the sequencing methods used in this study are described in Materials and Methods.

The Wisconsin State Laboratory of Hygiene (WSLH) processed biological duplicates of 129 wastewater samples. One replicate was concentrated using Nanotrap Microbiome A Particles (Ceres Nanosciences) and extracted with a Maxwell HT Environmental TNA kit (Promega) for Illumina WGS. The other replicate was concentrated using Nanotrap Microbiome A Particles and extracted with a MagMAX wastewater extraction kit (Thermo Fisher Scientific). At the University of Illinois, 210 wastewater samples were processed following the dPCR genotyping protocol. The purified SARS-CoV-2 RNA from these samples was tested using both dPCR and WGS. We evaluated the results from 339 combined samples processed by both methods.

### Concordance analysis

To synchronize the results, we aggregated WGS sublineages into broader lineages with shared mutations, aligning with the dPCR detection panels. Both positive and negative detections were considered. Concordance between the two methods ranged from 61.9% to 98.2% (Table 1).

**TABLE 1:** Concordance between dPCR-based genotyping and WGS for JN, EG.5, XBB, and FL variants^*a*^

Variant	No. in concordance	Percent concordance^*b*^
JN	333	98.2
EG.5	282	83.2
XBB	230	67.8
FL	210	61.9

^*a*^ A total of 339 wastewater samples were counted based on the detection or absence of the marker by each method.

^*b*^ Combined positive and negative percent agreement.

### Correlation analysis

**Fig 3:**
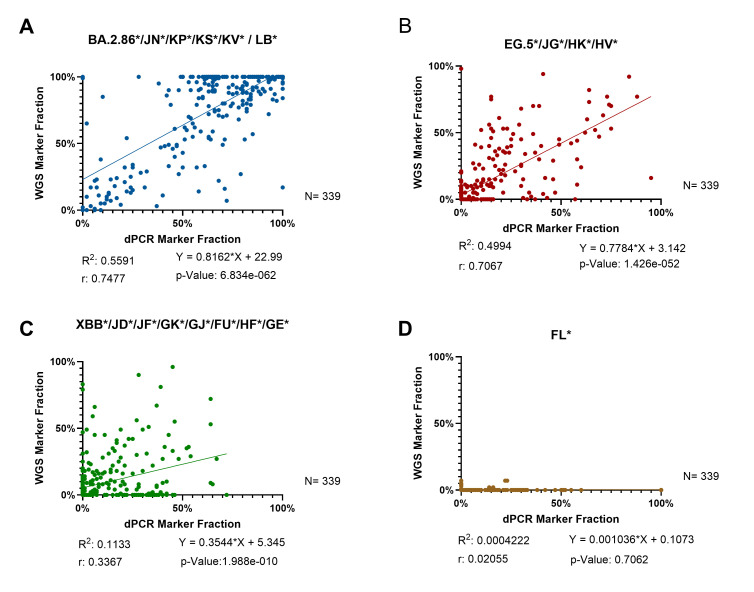
Correlation analysis between dPCR and WGS for 339 wastewater samples. (**A**) JN marker shows strong correlation and strong consistency. (**B**) EG.5 results demonstrate strong correlation and consistency. (**C**) XBB results demonstrate moderate correlation with higher variability. (**D**) FL results demonstrate no correlation.

The correlation analysis between dPCR and WGS results for JN, EG.5, XBB, and FL variants further supported the agreement between the findings and revealed varying degrees of consistency between the two methods (Fig. 3). For the JN variant, there was a strong positive correlation (*r* = 0.7477, *P* < 0.0001), indicating a high level of agreement between dPCR and WGS. Similarly, the EG.5 variant demonstrated a statistically significant positive correlation (*r* = 0.7067, *P* < 0.0001), reflecting strong alignment in detection results. In contrast, the XBB variant showed a moderate positive correlation (*r* = 0.3367, *P* < 0.0001), highlighting considerable variability and reduced agreement between the methods. For the FL variant, no significant correlation was observed (*r* = 0.02055, *P* = 0.7062), with notable differences in detection frequencies; WGS identified FL in only 3% of samples, compared to 37% in the dPCR results. These findings underscore differences in detection sensitivity, methodological resolution, and baseline detection levels between the two approaches, particularly for recombinant variants like XBB and FL.

### Lessons learned and opportunities for improvement

We encountered several challenges during this project, resulting in valuable lessons for future deployments of dPCR-based genotyping for wastewater, which we have summarized in Table 2.

**TABLE 2:** Lessons learned and opportunities for improvement^*a*^

Challenge	Potential improvement
The primer and probe design tools we used were not optimized for the dPCR system that we were using for our testing, which caused some of the primer and probe sets to fail when implemented on the dPCR system.	Utilize primer and probe design tools developed by the manufacturer of the dPCR instrument.
The assays that we used for genotyping were not available with customized concentrations of primers and probes, which caused some of the primer and probe sets to fail when implemented on the dPCR system.	Work with an assay manufacturer that provides customized concentrations of primers and probes, allowing for optimization specific to wastewater samples.
The JN variant rapidly grew in prevalence and was the dominant variant in samples before we had a JN assay validated and deployed to the testing labs, resulting in high levels of “unknowns” detected in the wastewater samples during this time period.	New variants can arise quickly and are expected to appear in wastewater samples earlier than in clinical samples. Having verified assays ready for deployment as soon as possible is desirable. This may require designing, ordering, and testing assays on a more frequent cadence.
Using a single-target mutation assay for each variant detection is not the most cost-effective or labor-efficient way to utilize dPCR testing.	Instead of single-target mutation assays, which require running four separate dPCR plates each week in the testing lab, use multiplex assays to reduce the burden on the testing laboratories. Based on our estimated costs for assays, controls, reagents, and plates, moving to a four-plex dPCR assay could reduce the cost per sample by $66.
The data analysis pipeline failed when the dPCR instrument manufacturer published a software update on the instrument. This update modified the data output format and caused issues during data upload to the ROSALIND Tracker.	Implement a QC check on data format in the data management process to prevent data upload/transfer issues. Coordinate with the instrument manufacturer to be prepared for pending software updates and the impact those changes might have on the process.
Overall concentrations of SARS-CoV-2 RNA in the wastewater samples were not reported in this project, potentially diminishing the value of this testing method.	Including an overall SARS-CoV-2 target in the test panel can be a useful metric for monitoring community transmission. Understanding virus levels alongside variant prevalence provides a better picture of transmission dynamics. Although this study did not measure SARS-CoV-2 levels in wastewater, it is possible to do so by analyzing mutant and wild-type quantities in each assay. Further study is needed to assess the accuracy of SARS-CoV-2 levels based on these results.

^*a*^ QC, quality control.

### Evaluating dPCR genotyping of wastewater at a national level

To evaluate the utility of, and any challenges associated with, utilizing this dPCR-genotyping method at a national level, we worked with multiple testing laboratories starting on 31 October 2023. The wastewater genotyping panel 2 for variants XBB, EG.1, EG.5, and FL was deployed by five laboratories around the United States. These laboratories, collectively, tested samples from six states (California, Georgia, Illinois, Louisiana, New York, and Wisconsin) at a cadence of roughly 17 samples per lab per week.

**Fig 4:**
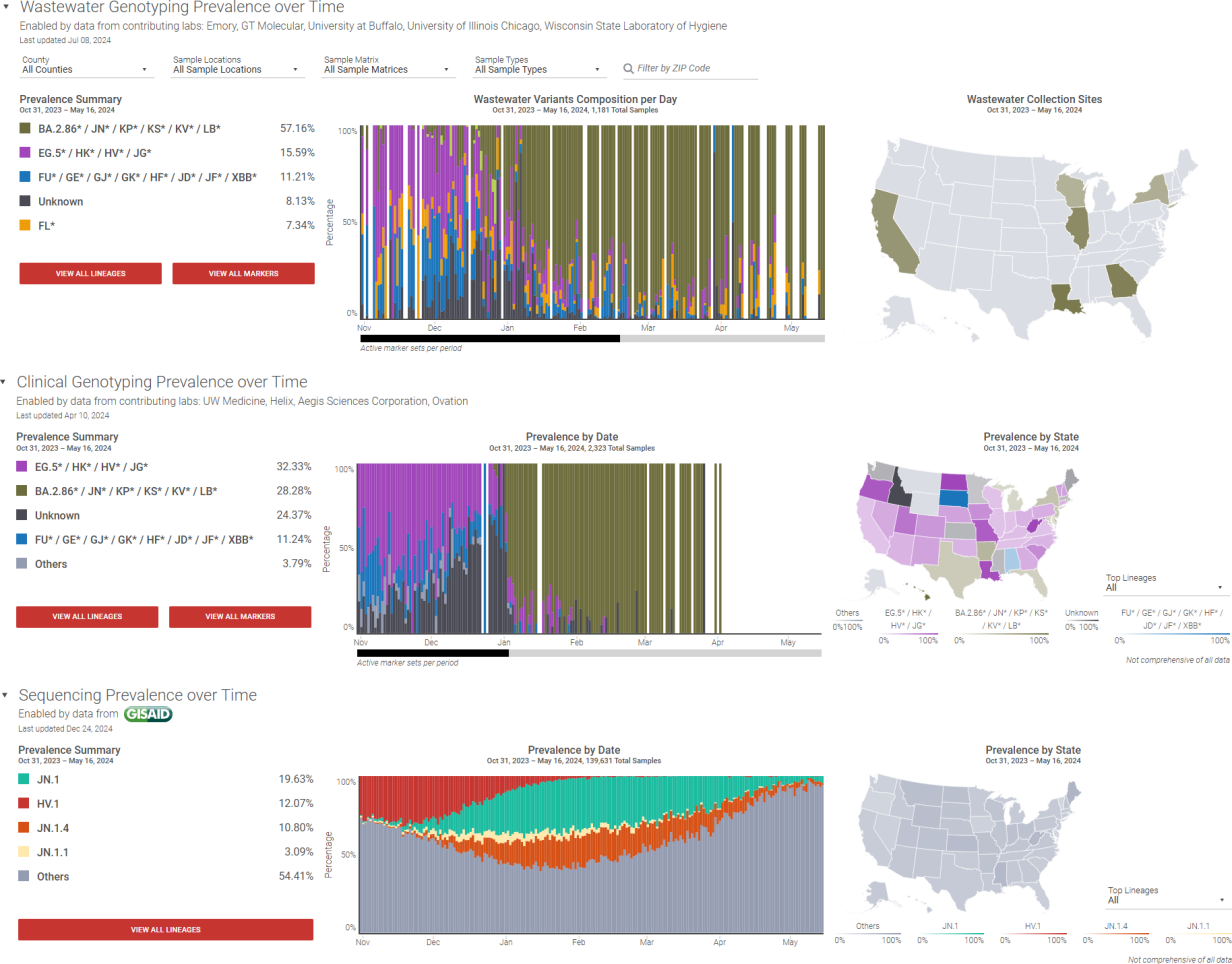
National comparison of SARS-CoV-2 variant prevalence across wastewater, clinical, and sequencing data sets (October 2023 to May 2024). Top: the wastewater genotyping dashboard illustrates the prevalence of SARS-CoV-2 variants in wastewater samples collected across six states. Middle: the clinical genotyping dashboard presents the variant prevalence from clinical samples across the United States. Bottom: GISAID sequencing dashboard displays sequencing data from the GISAID database, covering a wide range of SARS-CoV-2 variants. All three data sets indicate the steady rise of JN.1 and JN* variants starting in November 2023, replacing EG.5*/HV.1 variants and becoming dominant by early 2024. Note that only the top four lineages/variants in this period are listed. Snapshot image from ROSALIND Tracker (https://tracker.rosalind.bio/tracker/dashboard/).

Between 31 October 2023 and 16 May 2024, 1,181 samples from these six states were processed and analyzed using the dPCR genotyping approach (Fig. 4). During that same time frame, 2,323 clinical samples nationwide were analyzed for the same variants using qPCR-based genotyping, and 139,631 clinical sequencing results were deposited in GISAID. As observed in the detailed analysis of Georgia samples, the emergence of the JN.1 variant was detected in early November 2023 (Fig. 2). This variant showed a rapid rise in prevalence in combined wastewater data from these six states, increasing from a 2-week average of approximately 9% in December 2023 to around 75% in January 2024 in wastewater samples (Fig. 4). A similar pattern of rapid emergence was observed in the GISAID data.

From a broader perspective, the wastewater variant profiles in these six states during this period mirrored the patterns observed in the GISAID data set for the entire country. For example, the wastewater dPCR-based genotyping data showed a JN marker prevalence of 57.16% (dark green in Fig. 4), which closely aligns with the 61.31% observed in the GISAID sequencing-based data set. Note that the JN marker detected by the dPCR genotyping BA.2.86*/JN* assay corresponds to JN.1, JN.1.1, and JN.1.4 in the GISAID data set. Furthermore, the “Other” category in the GISAID graph includes 27.79% of BA.2.86 sublineages. This results in an overall prevalence of 61.31% for the JN marker in the GISAID data set for this period. This strong agreement highlights the reliability of dPCR-based wastewater genotyping as a complementary tool to clinical and sequencing-based approaches for variant monitoring.

## DISCUSSION

In this study, we present a novel approach for detecting SARS-CoV-2 variants in wastewater using dPCR and mutation-specific assays. Several studies have previously utilized mutation-specific PCR assays to detect SARS-CoV-2 variants of concern in wastewater (14, 16, 17, 21). However, these studies primarily used individual mutation-specific assays to track specific mutations, either retrospectively or in real-time, without the capability to detect emerging variants circulating in the community.

Compared to these approaches, our study employs panels of mutation-specific duplex assays with probe-specific mutation and wild-type probes, specifically designed for real-time wastewater surveillance of Omicron BA.2 sublineages, enhancing detection accuracy. Additionally, we introduce a novel prevalence calculation method for recombinant variants sharing mutations within our assay panel and demonstrate the ability to detect and measure the prevalence of emerging variants, making this approach valuable for near-real-time proactive surveillance. Moreover, our workflow includes an automated viral concentration and nucleic acid extraction process, improving efficiency and reproducibility.

Although the accuracy of wastewater testing can be affected by sewer shed-specific characteristics (22, 23), our 1-year study in the state of Georgia shows that the prevalence of variants detected in the wastewater closely matched the variant prevalence in clinical specimens, reinforcing the reliability of this approach as reported by other studies (12, 14, 16). A detailed analysis of multiple data sets from Georgia revealed several key findings. First, the dominance and transition of SARS-CoV-2 variants, such as the shift from XBB to BA.2.86*/JN* in early 2024, were effectively captured in both wastewater and clinical samples (Fig. 2). We also noted the clinical genotyping data tracked XBB’s decline until October 2023 when reporting ceased due to a sharp reduction in genotyped samples. The number of clinical specimens declined from 62 in early August to just 2 by late September 2023, and no clinical samples were genotyped by the reference labs after 8 February 2024. This decline in testing likely hindered accurate prevalence assessment in the qPCR-based clinical results, but with sufficient samples, the trendline would have likely mirrored the GISAID and wastewater genotyping results.

The data from Georgia indicate that some variants were transient, like BQ* and EG.1*. The brief spike in the BQ* variant detected in wastewater in May 2023, and the absence of a corresponding spike in clinical and GISAID data, raises questions about the possible reasons for this discrepancy. It may reflect a lag in clinical testing, differences in population sampling, or the variant’s low pathogenicity, leading to fewer clinical cases with specimen collection and genotyping despite widespread circulation. The detection of the EG.1* variant solely in the wastewater data during the fall and winter of 2023, with no corresponding signals in clinical or GISAID data sets, suggests that this variant did not lead to significant numbers of clinical cases.

We observed two significant spikes in the Unknown/Others category during the study: in June and October 2023. These spikes indicate ongoing viral evolution and the emergence of new variants. The June spike likely resulted from the introduction of the FL variant, which was reported in GISAID data but went undetected by the wastewater genotyping panel, as it did not include specific primers and probes for FL at that time (Fig. 2C). In October 2023, we suspected that the unknown variants detected were JN. Retesting archived nucleic acids from 23 wastewater samples collected between 11 December 2023 and 1 January 2024 confirmed this, as the unknown variants disappeared after deploying the JN assay (Fig. S13 and Fig. 2D). The concurrent rise of unknown and JN variants from October 2023 to April 2024 demonstrated strong alignment between wastewater and GISAID trendlines. However, the rapid rise of JN in late 2023 posed a challenge. We were unable to validate and deploy the assay across all five testing laboratories in time to track JN in real time. As a result, a large fraction of unknown variants appeared in wastewater samples tested during January and February 2024. After assay validation, laboratories retested retained RNA samples, and the updated results were uploaded to the ROSALIND Tracker. This highlights the need for more frequent assay design and validation to keep pace with emerging variants.

The concordance study between dPCR and WGS results for JN, EG.5, XBB, and FL variants in Wisconsin and Illinois demonstrates that dPCR genotyping is an accurate and reliable tool for monitoring variants in wastewater. These findings align with previous studies highlighting the higher sensitivity and lower sample input requirements of PCR-based methods (17, 24, 25). However, we noted varying degrees of agreement between dPCR and WGS variant prevalence results. The lower agreement for XBB and FL markers likely stems from differences in single-nucleotide polymorphism detection in dPCR and lineage classification protocols in WGS. Recombinant variants such as XBB—formed from multiple lineages—can carry mutations shared in other variants like FL (26) (https://covariants.org/variants/22F.Omicron; https://genspectrum.org/). These mutations are detected by dPCR, but they are classified under different categories in WGS, resulting in discrepancies between the two methods. Similarly, FL variant detection often requires multiple markers, introducing greater variability due to the additional measurements and percentage normalization steps. In contrast, the JN marker showed higher agreement due to the presence of unique mutations not shared with other variants, enabling consistent identification across methods. In addition, there are some limitations with sequencing-based genotyping when genome coverage is low, since errors in variant identification might occur due to only partial information on the analyzed RNA (17, 24). Despite detection sensitivity, variant nomenclature, and methodological resolution, dPCR genotyping offers a reliable approach for rapid and targeted variant monitoring, complementing the higher-resolution capabilities of WGS.

### Conclusion and recommendations

PCR-based genotyping offers a potential alternative to sequencing for rapid, cost-effective variant detection in wastewater. However, this study identified key challenges in implementing dPCR-based wastewater genotyping and proposed improvements. Assay failures occurred due to primer and probe design tools not being optimized for the dPCR system, which could be mitigated by using manufacturer-recommended design tools and customized primer concentrations. Single-target mutation dPCR assays proved inefficient and costly, suggesting a shift to multiplex assays. Additionally, a software update from the dPCR instrument manufacturer disrupted the data pipeline, highlighting the importance of proactive quality control measures and coordination with manufacturers. Lastly, the study did not measure overall SARS-CoV-2 concentrations in wastewater, limiting its utility for community transmission analysis. Future efforts should include an overall SARS-CoV-2 target to enhance surveillance capabilities.

Despite challenges, the dPCR genotyping method offers significant advantages over WGS for routine variant surveillance, including lower costs, simplicity, and faster turnaround times. As of 2024, singleplex dPCR reduced reagent costs by 36% and sample-to-result times by 68% compared to Illumina sequencing (Table S7). Multiplexing in dPCR could further cut reagent costs by 84% and processing times by 90%. The streamlined dPCR workflow requires fewer steps and less expertise, and it allows rapid marker updates without extensive revalidation, making it ideal for tracking evolving variants. While WGS provides greater precision and detects recombinants, dPCR is a practical, cost-effective choice for ongoing high-throughput SARS-CoV-2 wastewater surveillance. However, unlike WGS, PCR-based genotyping cannot identify novel mutations or provide comprehensive genomic information, making it less suitable for exploratory surveillance. Compared to qPCR, dPCR offers high sensitivity, accuracy, and resilience to inhibitors, but the run time is longer, and it may be less suitable for detecting multiple targets in a high-throughput format due to the cost and complexity of multiplexing (27, 28).

In our 1-year study in Georgia, we observed that one variant consistently exceeded 50% prevalence each quarter, with no more than three variants dominating the profile at any given time. This suggests that, for community-level surveillance programs, a broad detection tool like dPCR-based genotyping can provide sufficient resolution to monitor major shifts in viral variants and may be adequate for understanding clinical outcomes. High-resolution methods like WGS, which detect sublineages, might not always be necessary in such contexts. Furthermore, these findings emphasize the need for regularly developing and validating multiplexed wastewater panels to capture the continuously evolving variant landscape effectively. During the study, we also observed the presence and a sudden increase in the Unknown variants at different time points. Establishing a detection threshold for the Unknown marker and monitoring the rate of increase could serve as indicators to trigger new assay development and validation efforts. The ability to rapidly identify emerging variants is critical for timely public health interventions.

We compared the wastewater genotyping results from six states, representing the Northeast, South, Midwest, and West regions of the United States, to clinical qPCR-based genotyping data and sequencing results in GISAID. Despite the smaller regional coverage compared to nationwide data and the smaller sample size in the wastewater data set relative to clinical data sets, the comparison demonstrated similar trends and variant profiles. This strong agreement underscores the reliability of dPCR-based wastewater genotyping as a complementary tool to clinical specimen testing and sequencing-based approaches for monitoring SARS-CoV-2 variants.

Overall, our findings support the use of wastewater-based SARS-CoV-2 genotyping as a cost-effective and complementary approach to clinical sample genotyping and sequencing, offering a broader and more inclusive picture of variant prevalence and transmission. This approach is particularly valuable in times when clinical testing is limited, as observed during the study period. Beyond SARS-CoV-2, the dPCR-based genotyping platform described in this study holds promise for tracking other pathogens in wastewater. Its high sensitivity and specificity make it particularly suitable for monitoring low-abundance targets, such as emerging variants of other respiratory viruses (e.g., influenza and respiratory syncytial virus [RSV]), antibiotic resistance genes, or enteric pathogens like norovirus and *Salmonella*. As public health agencies expand their use of wastewater surveillance, dPCR can serve as a flexible and scalable tool for early detection and population-level monitoring of a wide range of infectious diseases.

## MATERIALS AND METHODS

### Wastewater collection

We processed and analyzed 1,416 wastewater samples across six states from April 2023 to May 2024. Table 3 lists the number of samples tested from each state and the collection period for those states.

**TABLE 3:** Summary of wastewater samples collected across six states^*a*^

State	Total wastewater samples	Start	End
California	155	10/31/2023	04/24/2024
Georgia	528	04/11/2023	04/05/2024
Illinois	322	10/31/2023	05/16/2024
Louisiana	101	02/27/2024	04/25/2024
New York	152	01/04/2024	04/25/2024
Wisconsin	158	11/22/2023	02/01/2024

^*a*^ Overview of the total number of wastewater samples collected, along with the start and end dates of sample collection, from each state. The data spans from 11 April 2023 to 16 May 2024, across California, Georgia, Illinois, Louisiana, New York, and Wisconsin. Dates are given as month/day/year.

Wastewater samples were collected from multiple types of sources, including wastewater treatment plants and correctional facilities, as part of regular wastewater testing routines conducted by the five testing laboratories. Wastewater samples were collected from 91 sites across six U.S. states using primarily automated composite sampling methods. Most sites employed 24-hour time- or flow-weighted composite sampling of influent wastewater from treatment plants. California sites collected effluent samples, and one site in Illinois provided grab samples. The sampling frequency ranged from once to twice weekly, and volumes collected per site ranged from 40 mL to 500 mL. Environmental monitoring data, including sample arrival temperature, total flow, and optionally pH, DO, and conductivity, were recorded where available—most comprehensively in Wisconsin and Illinois. Full sampling specifications by state, including site type, method, and environmental parameters, are provided in Table S8.

Across the six participating states, most wastewater samples were processed promptly upon arrival or stored under appropriate conditions to preserve sample integrity prior to analysis. When processing delays occurred, they were typically due to logistical factors such as sample shipping schedules or batching strategies for efficient lab workflow. More details are provided in the Supplemental material.

### Wastewater processing

Automated SARS-CoV-2 concentration was accomplished using Nanotrap Microbiome A Particles and Enhancement Reagent 1 (ER1) on a Thermo Scientific KingFisher Apex System. In brief, 75 µL of Nanotrap Microbiome A Particles and 50 µL of Nanotrap ER1 were mixed with ~5 mL of wastewater in two replicate wells with a total of 10 mL of wastewater for each sample. Concentrated viruses were lysed in 500 µL Microbiome Lysis Buffer at 56°C.

For all samples except the samples from WSLH that underwent WGS, the following nucleic acid extraction method was followed. After viral concentration, the samples were processed for RNA extraction using the Applied Biosystems MagMAX Wastewater Ultra Nucleic Acid Isolation Kit on the KingFisher Apex System. Briefly, 400 µL of lysate was mixed with 550 µL of MagMAX binding mix, and 10 µL of proteinase K was added to each sample prior to running on the KingFisher Apex. Wash 1 and wash 2 used 1 mL of MagMAX wash solution and 80% ethanol, respectively. RNA was eluted in 100 µL Microbiome Elution Solution. For the WSLH samples that underwent WGS, nucleic acids were extracted using the Maxwell HT Environmental TNA kit (Promega).

Extracted nucleic acids were quantified post-extraction and either analyzed via dPCR genotyping within 24 hours or were stored in a freezer at −20°C to −80°C until they were analyzed.

### SARS-CoV-2 dPCR-based genotyping

SARS-CoV-2 genotyping was accomplished using the Applied Biosystems TaqMan SARS-CoV-2 Mutation Panel (Cat# A49785). Table 4 summarizes the assays and targeted mutation sites used in this study.

**TABLE 4:** Assay and mutation sites for SARS-CoV-2 genotyping^*a*^

Assay ID	Mutation	Variants	Period
CV9HHWW	G28681T	BQ*	January 2023 to August 2023
CV32Z67	A19326G	XBB*	January 2023 to February 2024
CVXGPWV	C28928T	EG.1*	August 2023 to December 2023
CVU62R2	C29625T	EG.5*	January 2024 to February 2024
CVEPRZU	G5720A	FL*	April 2023 to February 2024
		EG*	June 2023 to March 2024
ANCFPZX	G8393A	JN*	December 2023 to May 2024
		KP*	March 2024 to May 2024
		LB*	April 2024 to May 2024

^*a*^ This table lists the assays used for detecting specific SARS-CoV-2 variants based on their mutations. Each row includes the ThermoFisher assay ID, the specific mutation it targets, and the variants and sublineages detected. For example, assay ANCFPZX targets the G8393A mutation and is used for identifying the JN variants. Panels of two or four assays were used in combination in order to monitor the prevalence of different SARS-CoV-2 variants. The genotyping panels changed over time to follow the virus mutations.

The marker selection for SARS-CoV-2 variant detection and lineage assignment methods used in this study has been previously described (15). Sequences for positive controls (mutation and wild type) were designed *in silico* by ROSALIND Bio and were constructed and manufactured using the gBlocks service by Integrated DNA Technologies (IDT) or the GeneArt service by ThermoFisher Scientific. Assays and controls were validated by Emory University and Ceres Nanosciences, Inc., using the QIAcuity dPCR system.

The mutation detection assay was performed on the QIAGEN QIAcuity Digital PCR system. In brief, the reaction mix was made by mixing 10 µL OneStep Advanced Probe Master Mix and 0.4 µL OneStep Advanced RT Mix. The volume was brought up to 30 µL by adding RNase-free water. Ten microliters of RNA template was added to the reaction mix, and then the entire volume of 40 µL was transferred into 26K 24-well QIAGEN Nanoplate. The QIAGEN QIAcuity Digital PCR system was used to amplify and detect the signals. Amplification was accomplished according to the following steps: (i) one cycle at 50°C for 40 minutes; (ii) one cycle at 95°C for 2 minutes; (iii) 45 cycles at 95°C, for 3 seconds; (iv) 60°C for 30 seconds. Only for the FL assay, step iii was modified to 45 cycles at 95°C for 30 seconds, and step iv was modified to 57°C for 1 minute. Signal detection was obtained using default settings for exposure duration and gain in each channel.

QIAcuity Software Suite (version 2.2) was used to analyze the data. A common threshold was applied across the samples to clearly separate negative partitions from positive partitions. Mutation detection results were exported in comma-separated values (CSV) format.

### Calculating variant percentages

Three genotyping panels were used to track SARS-CoV-2 variants as their prevalence shifted in the United States (Table S1 through S3). Each panel included 2–4 digital PCR assays targeting specific mutations and their wild-type counterparts. Mutation fractions were calculated as follows:


$$mutation\;fraction\;\%=\;\frac{mutant\;concentration}{mutant\;concentration\;+\;wild\;type\;concentration}\times 100.$$

Unique mutations identified specific lineages, while shared mutations required subtraction of overlapping fractions in a pre-defined order. Variant prevalence was normalized if the total exceeded 95%, ensuring the sum approached 100%. Undetected variants were calculated by subtracting the total mutation fraction from 100%. Detailed calculations are in Table S4.

### Data analysis and presentation on the public dashboard

Details of the controls and sample analysis criteria can be found in the Supplemental material. The ROSALIND classification algorithm automated mutation fraction analysis from QIAcuity dPCR outputs, processing thousands of specimens per minute on Google Cloud’s secure virtual private cloud. Metadata files with sample origin and collection dates were required for processing. Results were published on the publicly accessible ROSALIND Tracker dashboard (https://tracker.rosalind.bio/tracker/dashboard/).

### WSLH sequencing method

WSLH sequences roughly 20% of the wastewater samples it processes. Illumina WGS data are processed through the Viralrecon workflow (https://nf-co.re/viralrecon/). The bioinformatics algorithm Freyja is used to evaluate the relative proportion of the SARS-CoV-2 lineages present in wastewater samples. Data are manually curated to only display the lineages according to the World Health Organization (WHO) and Nextstrain nomenclatures. These data and visualizations are available on a dashboard accessible to the public (https://dataportal.slh.wisc.edu/sc2-ww-dashboard).

WSLH utilized a library prep method for sequencing using the following protocol. The SARS-CoV-2 libraries were prepared using the QIAseq DIRECT SARS-CoV-2 Enhancer kit using the Booster primers (Qiagen). Briefly, single-stranded viral RNA molecules were reverse transcribed into cDNA using hexaprimers. The SARS-CoV-2 genome was then specifically enriched using a SARS-CoV-2 primer panel. The panel consists of approximately 550 primers for creating 425 amplicons, covering the entire SARS-CoV-2 viral genome. Prior to sequencing, library quality was assessed using the QIAxcel Advanced System (Qiagen) and quantified by qPCR using the QIAseq Library Quant System kit (Qiagen). Libraries were sequenced on a MiSeq Illumina platform using MiSeq Reagent v2 (300 cycles) kits targeting a median coverage of at least 500× and at least 80% of the genome covered at 10×.

To assess variant proportions, WGS data were analyzed using Freyja v.1.3.11, a tool specifically designed to estimate SARS-CoV-2 variant proportions in deep sequence data containing mixed populations (29). BAM files, generated using viralrecon v2.5, were processed through Freyja, utilizing the Wuhan-Hu-1 reference genome (MN908947.3) to produce variant and depth files. The median estimates were obtained through Freyja’s bootstrap boot function (nb = 10). All samples were processed using Freyja’s UShER barcode reference database updated on 13 April 2024. This ensured the inclusion of all the most recent variant detections in samples processed during earlier periods.

Variant proportions derived from Illumina sequencing are accessible through a publicly available dashboard hosted at https://dataportal.slh.wisc.edu/sc2-ww-dashboard. This dashboard showcases the proportions of major variant groups listed on https://covariants.org/, with estimations generated using Freyja’s raw calculations. Additional details on the methodology can be found at https://github.com/wslh-ehd/sc2_wastewater_data_analysis.

### University of Illinois Chicago sequencing method

The SARS-CoV-2 libraries prep, sequencing, and data analysis were similar to the WSLH method, except that the libraries were sequenced on an Illumina Nextseq 2000 using NextSeq 1000/2000 P1 Reagents (300 Cycles) kits, generating a median of 800,000 reads per sample. All samples were processed using Freyja’s UShER barcode reference database and updated weekly during the testing period to ensure the inclusion of the most recent variants detected in samples in real time.

## Data Availability

The dPCR-based genotyping data and GISAID-derived data sets used in this study are available through the ROSALIND Tracker (https://tracker.rosalind.bio/). Raw sequencing data have been deposited into the NCBI repository under the BioProjects PRJNA889839 (WSLH sequencing method) and PRJNA989260 (University of Illinois Chicago sequencing method).

## SUPPLEMENTAL MATERIAL

Supplemental material msystems.00229-25-s0001.docx Additional experimental details: workflow development, validation, and implementation.

ASM does not own the copyrights to Supplemental Material that may be linked to, or accessed through, an article. The authors have granted ASM a non-exclusive, world-wide license to publish the Supplemental Material files. Please contact the corresponding author directly for reuse.
