# Supplementary material for: A 1-year study on SARS-CoV-2 variant shifts in wastewater using dPCR: comparison with clinical and GISAID data
Source: mSystems. 2025 Oct 22;10(11):e00229-25. doi: 10.1128/msystems.00229-25 (PMC12625748; doi:10.1128/msystems.00229-25)
Supplement: Supplemental material — Additional experimental details: workflow development, validation, and implementation. [file msystems.00229-25-s0001.docx]

# Supplementary Information

## Project Overview and Timeline

The wastewater processing and dPCR genotyping workflow involves four steps: sample collection, automated viral concentration and nucleic acid extraction, dPCR analysis, and data visualization. Wastewater samples are initially collected from influent lines at treatment facilities. These samples are processed using Nanotrap Enhancement Reagent 1 (ER1) and Nanotrap Microbiome A Particles coupled with MagMAX™ Wastewater Ultra Nucleic Acid Isolation Kit, which are added to an automated machine (KingFisher Apex) to concentrate, purify, and extract RNA. The RNA samples undergo digital RT-PCR analysis to detect and quantify SARS-CoV-2 RNA. The resulting data is visualized to show the composition of different SARS-CoV-2 variants over time and their location, with a color-coded bar chart representing daily variant composition and a prevalence summary detailing the percent abundance of various identified lineages and markers. This workflow facilitates the monitoring and analysis of SARS-CoV-2 variants in the community through wastewater surveillance.

The project began in January 2023, focusing on adapting previously reported qPCR-based SARS-CoV-2 genotyping assays in clinical samples to wastewater for BA.1 and BA.2. This was followed by validating genotyping assays in the dPCR system (BQ.1 and XBB). The SOP for Panel 1 (BQ.1 and XBB) was released in April 2023. Validation for new assays for EG, FL, and FD variants began in June 2023. In November 2023, the SOP for Panel 2 (EG.1, EG.5, FL, XBB) was released, and four new testing labs started testing wastewater samples, expanding the testing sites to six states. In December 2023, validation of new assays for HV and JN variants was conducted, culminating in the release of the SOP for Panel 3 (JN, EG.5, FL, XBB) by February 2024. Figure S1 provides a timeline of project.

**
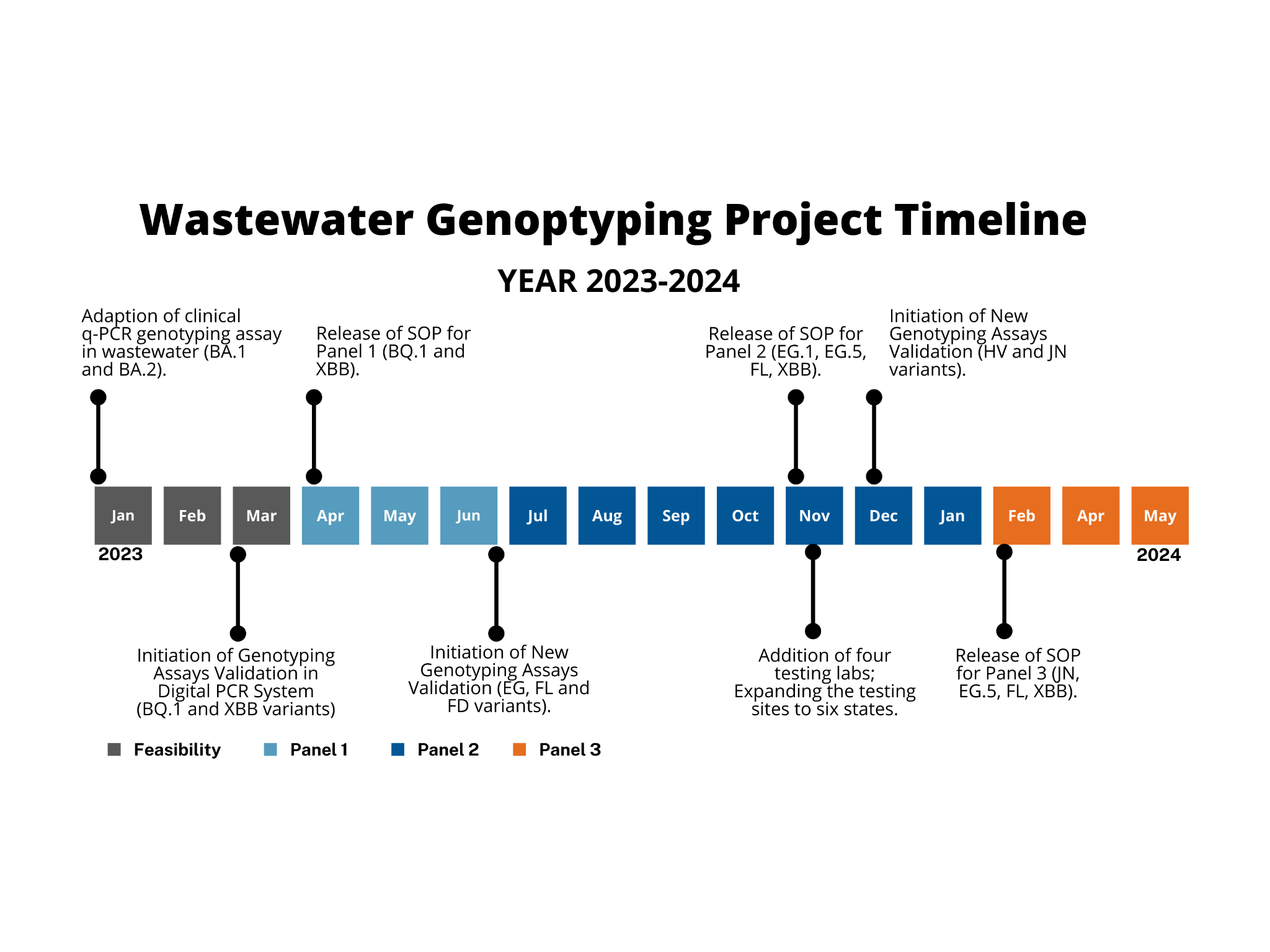
**

**Figure S1: Study Timeline for the dPCR genotyping project outlining key milestones in the development and validation of genotyping assays as well as panels implementation.**

Figure S2 illustrates the evolutionary relationships of SARS-CoV-2 variants as defined by Nextstrain clades. Starting from the 20B (B.1.1) lineage, it traces the progression and diversification of variants, including significant nodes like 21M (Omicron, B.1.1.529), which branches into sub-lineages such as 21K (Omicron, BA.1) and 21L (Omicron, BA.2). The genotyping assays used in this project targeted several variants in the 21L (Omicron, BA.2) clade such as 23I (BA.2.86) and 22F (XBB), as well as a variant in the 21K (Omicron, BA.1) clade. The tree uses color coding to differentiate between major variants and sub-variants.


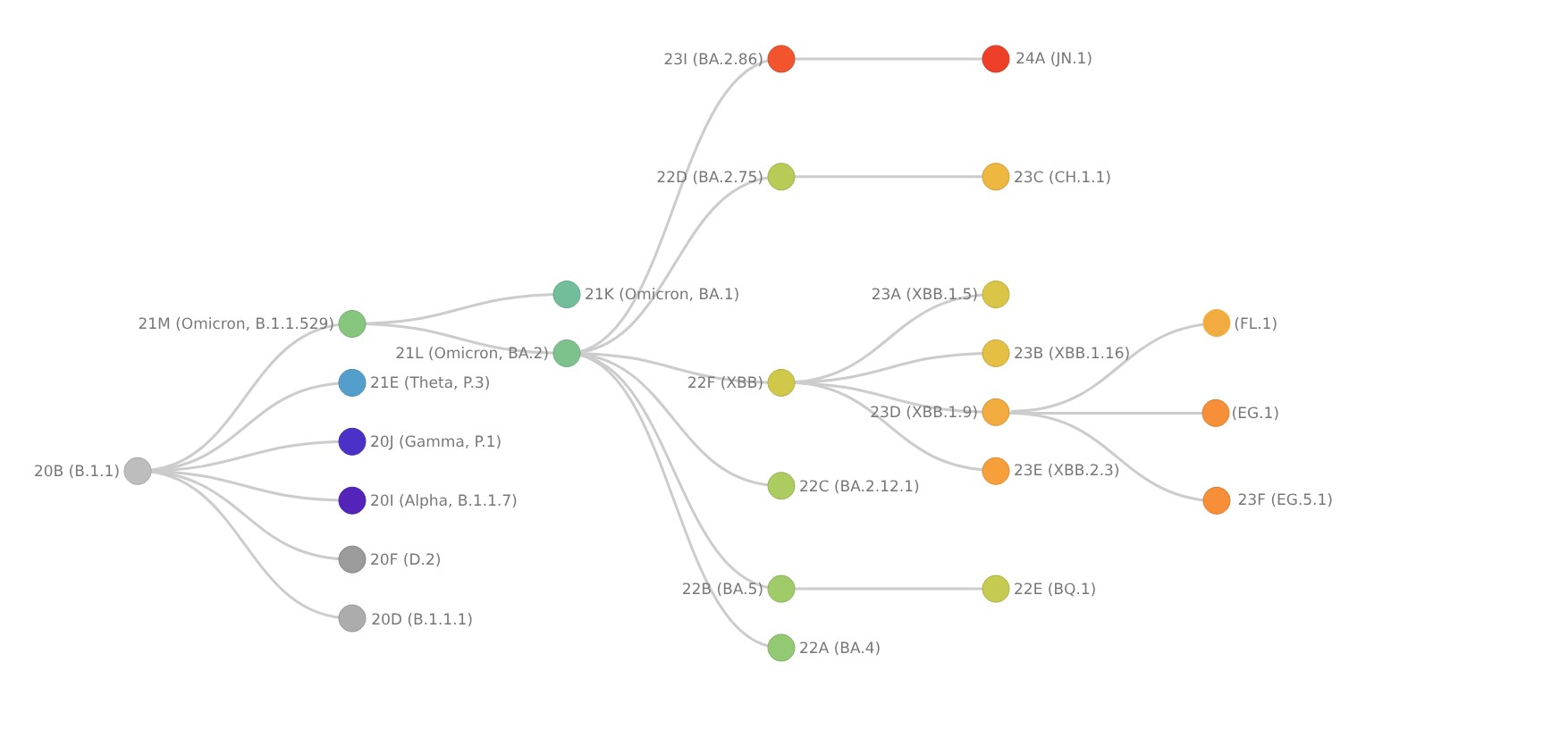


## **Figure S2: Phylogenetic Tree of SARS-CoV-2 Variants.** This figure illustrates the evolutionary relationships between SARS-CoV-2 variants as defined by Nextstrain clades. Genotyping assays in Table 6 target several variants in the 21L clade. The phylogenetic tree was adapted from [Nextstrain's ncov-clades-schema](https://github.com/nextstrain/ncov-clades-schema) with some modifications (<https://github.com/nextstrain/ncov-clades-schema>) (<https://nextstrain.org/>).

## SARS-CoV-2 dPCR Variant Genotyping Panels

Three variant genotyping panels were utilized, with new panels deployed as the relative prevalence of different SARS-CoV-2 variants changed in the United States (Table S1, S2, S3).

**Table S1: Panel 1 SARS-CoV-2 Variant Classification Based on Mutations (April 11, 2023 - June 19, 2023).** Presence of G28681T mutation identifies Omicron BQ* variant, while A19326G mutation identifies Omicron XBB* variant. N/A indicates mutations not applicable.

| **April 11, 2023 - June 19, 2023** | | | | |
| --- | --- | --- | --- | --- |
| **A19326G** | **C3857A** | **G26529A** | **G28681T** | **Variants Detected** |
| N/A | N/A | N/A | **✔** | Omicron BQ* |
| **✔** | N/A | N/A | N/A | Omicron XBB* |

**Table S2: Panel 2 classification of SARS-CoV-2 Variants by Mutations (June 21, 2023 - February 18, 2024).** The table classifies SARS-CoV-2 variants based on the presence (✓) or absence (✗) of specific mutations. Variants are classified as EG.1* with mutations A19326G, C28928T, and G5720A, FL* with mutations A19326G and G5720A, XBB*/JD*/JF*/GK*/GJ*/FU*/HF*/GE* with mutation A19326G, and EG.5*/JG*/HK*/HV* with mutations A19326G, C29625T, and G5720A.

| **June 21, 2023 - February 18, 2024** | | | | |
| --- | --- | --- | --- | --- |
| **A19326G** | **C29625T** | **C28928T** | **G5720A** | **Variants Detected** |
| **✔** | **X** | **✔** | **✔** | EG.1* |
| **✔** | **X** | **X** | **✔** | FL* |
| **✔** | **X** | **X** | **X** | XBB*/JD*/JF*/GK*/GJ*/  FU*/HF*/GE* |
| **✔** | **✔** | **X** | **✔** | EG.5*/JG*/HK*/HV* |

**Table S3: Classification of SARS-CoV-2 Variants by Mutations on Panel 3 (February 19, 2024 – May 16, 2024).** BA.2.86*/JN* identified by G8393A; FL* by A19326G and G5720A; XBB*/JD*/JF*/GK*/GJ*/FU*/HF*/GE* by A19326G; EG.5*/JG*/HK*/HV* by A19326G, C29625T, and G5720A.

| **February 19, 2024 – May 16, 2024** | | | | |
| --- | --- | --- | --- | --- |
| **A19326G** | **C29625T** | **G8393A** | **G5720A** | **Variants Detected** |
| **X** | **X** | ✔ | **X** | BA.2.86*/JN*/KP*/KS*/  KV* / LB* |
| ✔ | **X** | **X** | ✔ | FL* |
| ✔ | **X** | **X** | **X** | XBB*/JD*/JF*/GK*/GJ*/  FU*/HF*/GE* |
| ✔ | ✔ | **X** | ✔ | EG.5*/JG*/HK*/HV* |

The mutation detection assay was performed on the QIAGEN QIAcuity Digital PCR system. In brief, the reaction mix was made by mixing 10 μL OneStep Advanced Probe Master Mix and 0.4 μL OneStep Advanced RT Mix. The volume was brought up to 30 μL by adding RNase-free water. Ten μL of RNA template was added to the reaction mix, and then the entire volume of 40 μl was transferred into 26K 24-well QIAGEN Nanoplate. The QIAGEN QIAcuity Digital PCR system was used to amplify and detect the signals. Amplification was accomplished according to the following steps: 1- One cycle at 50°C for 40 minutes; 2- One cycle, at 95°C for 2 minutes; 3- Forty-five cycles, at 95°C, for 3 seconds; 4- 60°C for 30 seconds. Only for the FL assay, step 3 was modified to forty-five cycles, at 95 °C, for 30 seconds, and step 4 was modified to 57 °C for 1 minute. Signal detection was obtained using default settings for exposure duration and gain in each channel.

QIAcuity Software Suite (ver 2.2) was used to analyze the data. A common threshold was applied across the samples to clearly separate negative partitions from positive partitions. Mutation detection results were exported in CSV format.

## Calculating variant percentages

Each genotyping assay was designed to detect a specific single nucleotide mutation and the corresponding wild-type sequence in the SARS-CoV-2 genome. Using digital PCR, the mutation fraction was calculated using the formula below:

$$Mutation Fraction \%= \frac{Mutant Concentration}{Mutant Concentration + Wild Type Concentration}\times100$$

As SARS-CoV-2 evolved, combinations of mutations led to the emergence of different lineages and variants. For instance, the XBB variant is a recombinant lineage derived from the BA.2.10.1 and BA.2.75 sublineages of Omicron and includes the A19326G mutation (1) (<https://covariants.org/variants/22F.Omicron>). Another example is the EG.5 variant, which has acquired a set of mutations, including A19326G from XBB and C29625T, illustrating how SARS-CoV-2 can accumulate advantageous mutations from various lineages to potentially enhance its survival and spread (<https://covariants.org/variants/23F.Omicron>).

To monitor the dynamics of SARS-CoV-2 variants in wastewater, we tracked specific mutations using a combination of genotyping assays. This strategy enables the detection of unique mutations associated with specific lineages through direct detection. If a mutation is shared among multiple variants, the mutation fraction was determined by subtracting the fractions of other mutations. These adjustments were made in a pre-defined order to estimate the fraction of each variant detected in the wastewater samples. The sum of variant prevalence is expected to be close to 100% in each sample. To account for assay performance differences, we applied a normalization step if the total value was at or above 95%. For example, if the total prevalence was 97%, each variant prevalence was normalized by dividing by 0.97. Additionally, if there were undetected variants in the sample, the total prevalence will be below 95%. The percentage of undetected variants was obtained by subtracting the total mutation fraction in the panel from 100%.

Table S4 illustrates the SNP assays and associated lineages in panel 2. Assay 4 and assay 3 uniquely target the EG.5 and EG.1 lineages, respectively. Assay 2 detects the G5720A mutation, common to the FL, EG.1, and EG.5 lineages within this panel. To calculate the mutation fraction for the FL variant, we subtracted the EG.1 and EG.5 mutation fractions from the result of assay 2. Similarly, assay 1 targets mutation A19326G, which is shared by all four variants in this panel. By estimating the mutation fractions of EG.5, EG.1, and FL variants from the outcomes of assays 4, 3, and 2 respectively, we could then determine the XBB mutation fraction by subtracting these fractions from the result of assay 1. Table S4 shows an example of mutant fraction and variant fraction calculated using a mock wastewater sample in panel 2.

**Table S4: Mutation Detection and Fraction Analysis for SARS-CoV-2 Variants.** This table shows the detection of specific mutations in various SARS-CoV-2 lineages using different assays in Panel 2. Assay 3 (C28928T) and Assay 4 (C29625T) are specific to EG.1 and EG.5, respectively. The first step is to calculate the FL variant fraction by subtracting the results of Assays 3 and 4 from Assay 2. Following this first step, the XBB variant fraction is calculated by subtracting the results of Assays 2 (now corrected from previous step), 3, and 4 from Assay 1. For example, the Assay 1 (A19326G) has a mutant concentration of 70 Cp/μL and a wild-type concentration of 100 Cp/μL, resulting in a 41% mutant fraction and a 3% adjusted variant fraction after subtracting the contributions of Assays 2, 3, and 4 (38%). The unknown variant fraction is calculated by subtracting the combined known fractions from 100%. In this example, the unknown variant(s) show a notably high adjusted variant fraction of 59%.

| **Panel 2** | **Example of Genotyping Assay Result** | | | **Assigned Lineage/Variant** | | | | |
| --- | --- | --- | --- | --- | --- | --- | --- | --- |
|  | **Mut (Cp/µL)** | **WT (Cp/µL)** | **Mutant Fraction** | **XBB** | **FL** | **EG.1** | **EG.5** | **Unknown** |
| **Assay 1 (A19326G)** | 70 | 100 | *41%* | ✓ | ✓ | ✓ | ✓ | NA |
| **Assay 2 (G5720A)** | 60 | 100 | *38%* | ⨯ | ✓ | ✓ | ✓ | NA |
| **Assay 3 (C28928T)** | 20 | 100 | *17%* | ⨯ | ⨯ | ✓ | ⨯ | NA |
| **Assay 4 (C29625T)** | 10 | 100 | *9%* | ⨯ | ⨯ | ⨯ | ✓ | NA |
| **Variant Fraction** | | | | **3%** | **12%** | **17%** | **9%** | **59%** |

For multiple states, we compared the aggregated surveillance data on wastewater dPCR-based genotyping, clinical qPCR-based genotyping, and GISAID clinical sequencing-based genotyping, all of which were presented on the ROSALIND Tracker Dashboard. It is worth noting that, in contrast to clinical samples, which usually have one dominant variant (e.g., 100% XBB), the genotyping and sequencing results from wastewater typically present a profile of multiple variants (e.g., 70% JN, 20% XBB, 10% EG5) (2). This is because wastewater samples are composites with contributions from multiple individuals, capturing a broader range of circulating variants. Therefore, to calculate the variant prevalence, we aggregate the observed variant in each wastewater sample in two-week cadence and then the percentage of the total was calculated. On the other hand, typically only individuals with symptoms visit clinics for testing, so the variant prevalence in clinical samples is based on the number of patients observed with the variant of interest during the two-week period, relative to the total number of patients.

A classification algorithm was developed by [ROSALIND](https://www.rosalind.bio/) to automate the mutation fraction analysis in each sample based on the input from dPCR CSV files and assigned panels. A dedicated system was established to host the classification algorithm and to provide a web application with an application programming interface for standardized data submission and processing. This system was deployed on a secure virtual private cloud instance on Google Cloud Platform, enabling the processing of thousands of specimens per minute.

## Georgia Wastewater Samples

The Center for Global Safe Water, Sanitation, and Hygiene (CGSW) laboratory at Emory University was the first wastewater testing laboratory to use the dPCR-based wastewater genotyping method. Between April 2023 to April 2024, CGSW analyzed over 500 wastewater samples from Georgia, spanning three distinct genotyping panels referred to as “Panel 1”, “Panel 2”, and “Panel 3”.

During the feasibility phase, CGSW tested a combination of grab, Moore swabs, and composite samples from various locations such as public schools, influent lines at wastewater treatment plants (WWTPs), a county jail, and an airport. This breadth of sample types was utilized to assess the feasibility of dPCR-based wastewater genotyping across diverse locations to evaluate the method’s reliability and robustness.

After the feasibility phase, CGSW transitioned from testing multiple community sites to sampling selected WWTPs in metro Atlanta, as well as selected WWTPs throughout the state participating in the GA National Wastewater Surveillance System (NWSS) program. Testing was exclusively conducted on untreated composite wastewater samples sent to the Georgia Department of Public Health (GDPH) weekly from April 2023 to April 2024 and wastewater samples from metro Atlanta WWTPs collected by the CGSW collection team. Wastewater samples were stored at 4°C before concentration and extraction of SARS-CoV-2, as described in the wastewater processing method section. Extracted RNA samples were preserved at -20°C until dPCR analysis was performed for SARS-CoV-2 variant detection.

Figure S3 depicts the location and population size served by the wastewater collection sites and the total number of wastewater samples tested for Panel 1, Panel 2, and Panel 3 from April 2023 to April 2024. A total of 528 wastewater samples were tested from these WWTP sites in this period. The wastewater collection sites cover more than two million Georgia residents or approximately 18% of the state’s population. Most of the collection sites were in the Atlanta metropolitan area, reflecting the dense population and urbanization in Fulton, Gwinnett, and Cobb counties. Conversely, rural regions like Dougherty, Laurens, and Treutlen counties feature smaller facilities, corresponding to their sparse populations. The dispersed collection sites throughout various geographical locations ensured extensive coverage of the state of Georgia.


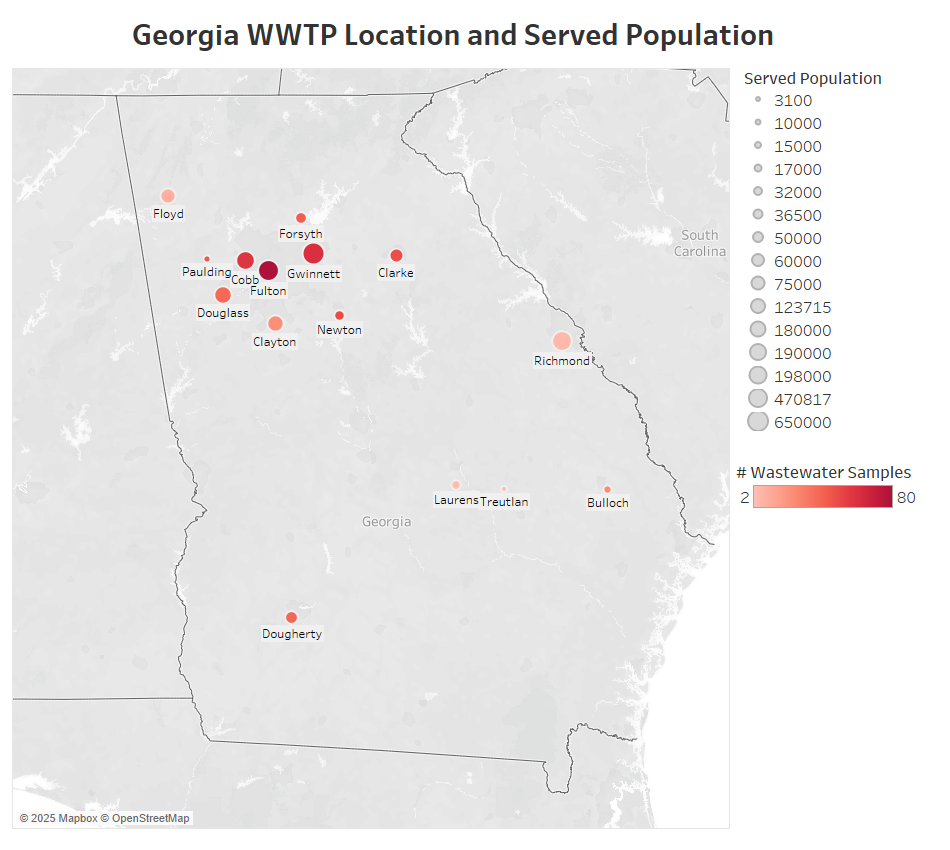


**Figure S3: Locations and Population Served by Wastewater Treatment Plants (WWTP) in Georgia.** This map illustrates the distribution of 17 Wastewater Treatment Plants (WWTP) across Georgia counties (3 WWTPs in Fulton county) and the populations they serve. The size of each circle represents the served population, with larger circles indicating larger populations. The color intensity, ranging from light to dark red, denotes the number of wastewater samples tested at each location, with darker shades representing higher sample counts. Key areas with significant populations and multiple facilities include Cobb, Fulton, and Gwinnett counties.

## Archived nucleic acid samples demonstrate feasibility of qPCR-based wastewater SARS-CoV-2 genotyping

First, we assessed the viability of using the pre-existing ROSALIND qPCR-based genotyping approach (which was being utilized to conduct genotyping for clinical samples) to wastewater samples by analyzing archived nucleic acid extracts from wastewater samples previously processed utilizing the Nanotrap Particle method as well as various variant homogenous and heterogenous controls.

Ten archived nucleic acid extracts from processed wastewater samples that were collected in a time frame that represented an expected transition from the BA.1 to the BA.2 lineage (between mid-February 2022 and mid-April 2022) were tested for BA.1 and BA.2 lineages using RT-qPCR genotyping assays (Cat# A49785) on QuantStudio according to the manufacturer’s recommendations. The BA.1 and BA.2 fractions in the wastewater samples were calculated using the ROSALIND automated pipeline originally developed for clinical samples. The same wastewater samples were also analyzed by NGS sequencing. Both methods consistently detected BA.1 and BA.2, with BA.1 prevalence decreasing and BA.2 increasing over time, becoming the dominant variant by early April 2022 (Figure S4). The "Unknown" category in qPCR genotyping and the "Others" category in NGS, present briefly in February and March, respectively, disappear by April, indicating improved detection and classification accuracy or suggesting the presence of other variants or sequencing artifacts that are later resolved. Statistical analysis of BA.1 and BA.2 showed p-values of 0.9269 and 0.1983, respectively, in a paired t-test, indicating no significant difference between dPCR genotyping results and NGS sequencing. Additionally, correlation coefficients (r) of 0.6514 and 0.9922, respectively, demonstrated a strong correlation between the results from the two methods. These findings support the feasibility of using qPCR-based genotyping for monitoring SARS-CoV-2 variants in wastewater.


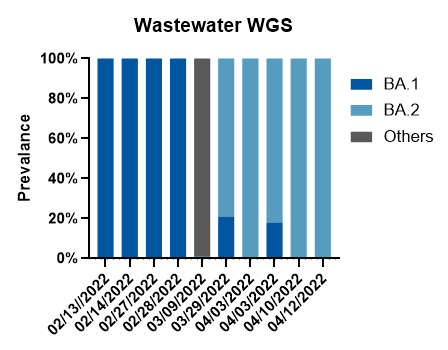

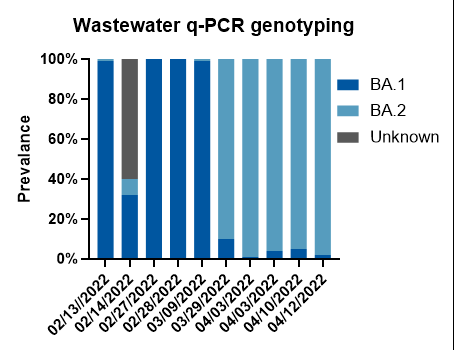


**Figure S4: Comparison of SARS-CoV-2 variant prevalence in wastewater samples using qPCR genotyping (top) and WGS (bottom) methods.** Both methods consistently detected BA.1 and BA.2 lineages from mid-February to mid-April 2022, showing a decrease in BA.1 and an increase in BA.2 prevalence over time. The results demonstrate the robustness and reliability of both techniques for monitoring SARS-CoV-2 variants in wastewater.

## Emory University (Georgia) Sequencing Method

For wastewater samples processed during the feasibility phase at Emory University, samples were also sequenced using the following protocol: Selected SARS-CoV-2 positive RNA samples were converted into cDNA using the SuperScript™ IV First-Strand Synthesis System and amplified with the ARTIC V4.1 nCOV-2019 Amplicon Panel kit, which uses 98 primers in two pools to detect mutations. Two separate PCR reactions were performed on each cDNA sample to ensure comprehensive amplification, followed by purification and preparation of NGS libraries. Low-quality libraries (≤20 ng/µL) were excluded. SARS-CoV-2 positive NGS libraries were sequenced using the NovaSeq6000 system, targeting 1 million reads per sample. Sequencing data was aligned to the Wuhan-Hu-1 genome and processed to remove ARTIC primer sequences, with insufficient depth samples excluded. The Freyja pipeline calculated the relative abundance of SARS-CoV-2 lineages, and variants detected at less than 0.01% were not reported. Lineages were categorized into Pangolin variants of concern, with rare variants

## Adapting the qPCR-genotyping method to a dPCR-genotyping method

Next, we utilized BQ.1 and XBB SARS-CoV-2 variant assays (Cat # CV9HHWW and Cat # CV32Z67, respectively) on the QIACuity Digital PCR System to evaluate the feasibility of using relative quantitation to determine the presence of variants in wastewater during both unique and transition variant periods. For dPCR, we relied on the proportions calculated by the dPCR software and made necessary adjustments as explained in the "Calculating Variant Percentages'' section. The ROSALIND team designed a new pipeline to calculate variant prevalence from dPCR files. The variant fractions in the qPCR method were calculated using the ROSALIND automated pipeline, originally developed for clinical samples and adapted for wastewater use (3). While clinical samples typically contain one lineage per sample, co-infections were observed regularly, allowing us to see the proportion of each variant in patient samples. Based on this, we updated our clinical qPCR pipeline to work with wastewater samples and predict the proportion of the main variants in the mix. To achieve this, in addition to the 100% wild type and 100% mutant controls, we added mixtures of these controls to correctly calibrate our proportion measurements. Figure S5 summarizes the genotyping results for nine wastewater samples collected between mid-January 2023 and early March 2023 using both methods.


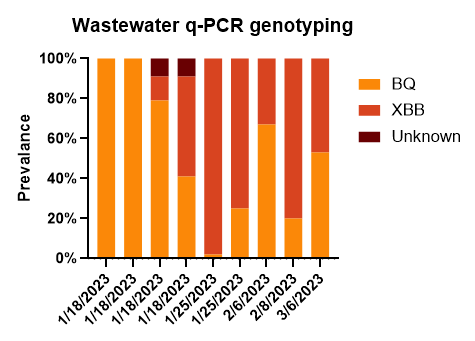


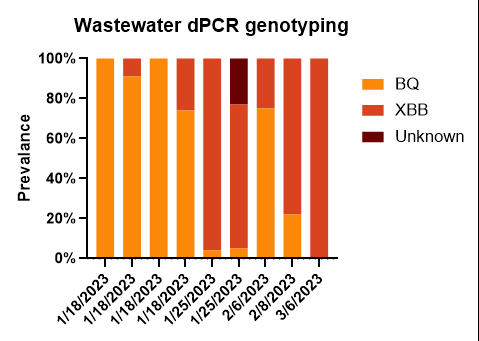


**Figure S5: Comparison of SARS-CoV-2 variant prevalence in wastewater samples using qPCR genotyping (top) and dPCR genotyping (bottom) methods for nine samples collected between January to March 2023.** Both methods consistently detect BQ (yellow) and XBB (orange) variants, with similar patterns of prevalence across samples. A transition from BQ to XBB variants is observed during this period. The results demonstrate the robustness and reliability of both qPCR and dPCR techniques for monitoring SARS-CoV-2 variants in wastewater.

The data shown in the lower image of Figure S5 depicts the relative amount of XBB and BQ.1 SARS-CoV-2 variants present in each wastewater sample as determined using the dPCR assay. The relative amount of XBB approaches 100% over the time period. In contrast, the relative amount of BQ.1 decreases to nearly 0% over the time period. Statistical analysis of BQ.1 and XBB showed p-values of 0.8342 and 0.8685, respectively, in a paired t-test, indicating no significant difference between qPCR and dPCR genotyping results. Additionally, correlation coefficients (r) of 0.8256 and 0.8537, respectively, demonstrated a strong correlation between the results from the two methods. These findings support the feasibility of using dPCR-based genotyping for monitoring SARS-CoV-2 variants in wastewater.

Wastewater samples collected between mid-February 2022 and mid-April 2022 showed a rapid displacement of BA.1 with BA.2, and samples collected between mid-December 2022 and early-March 2023 demonstrated a clear transmission from BQ.1 to XBB. These sub-lineage displacements detected by PCR assays were concordant with these sub-lineage dynamics in clinical samples reported in the literature (4-6). These results suggest that dPCR and RT-qPCR based assays can be used for specific and timely detection and monitoring of SARS-CoV-2 variants in wastewater. The slight variations in the proportion of variants detected by each method might be attributable to differences in sensitivity and specificity between qPCR and dPCR.

Data from the qPCR SARS-CoV-2 genotyping feasibility experiments with wastewater samples collected from within the Atlanta metro area demonstrated the utility of the method for the determination of XBB and BQ.1 relative amounts where SARS-CoV-2 N-gene qPCR assay threshold cycles (Ct) ranged from 30 – 37, which is within the typical range for circulating SARS-CoV-2 in wastewater samples (Table S5). A 37 Ct threshold was determined to be the recommended threshold requirement for the genotyping assays. Addition of controls at 0%, 25%, 50%, 75% and 100% were evaluated and it was determined that 0%, 50% and 100% are required for relative variant quantitation.

**Table S5: Performance of genotyping assay for dPCR at low, middle, and high Ct value.** This table presents the average N₁ real-time qPCR estimated Cₜ values alongside the detection results of XBB and BQ.1 variants using dPCR in eight wastewater samples. The data includes total positive partitions (MT+WT) and prevalence for both variants, highlighting varying detection levels across different Cₜ ranges. XBB variants were consistently detected at varying fractions across all samples, while BQ.1 variants showed much lower or zero detection rates in most samples.

| **Sample Label** | **Avg. N₁ Real-time qPCR** | **XBB dPCR** | | **BQ.1 dPCR** | |
| --- | --- | --- | --- | --- | --- |
|  |  | **Total Positive Partitions (MT+WT)** | **Variant Fraction** | **Total Positive Partitions (MT+WT)** | **Variant Fraction** |
| Wastewater 1 | Estimated Cₜ ~30-31 (Low Cₜ) | 18 | 100% | 23 | 0% |
| Wastewater 2 | Estimated Cₜ 32-33 (Middle Cₜ) | 7 | 100% | 7 | 0% |
| Wastewater 3 | Estimated Cₜ 32-33 (Middle Cₜ) | 13 | 53% | 10 | 40% |
| Wastewater 4 | Estimated Cₜ 32-33 (Middle Cₜ) | 10 | 90% | 4 | 0% |
| Wastewater 5 | 35.41 (High Cₜ) | 4 | 100% | 0 | 0% |
| Wastewater 6 | 35.79 (High Cₜ) | 11 | 67% | 2 | 33% |
| Wastewater 7 | Estimated Cₜ 36-37 (High Cₜ) | 8 | 100% | 5 | 0% |
| Wastewater 8 | 36.89 (High Cₜ) | 1 | 83% | 5 | 17% |

## Assessment of both 96-well and 24-well microwell plate formats on QIACuity dPCR system

The SARS-CoV-2 genotyping assays manufactured by ThermoFisher were validated on QuantStudio qPCR Instruments. Compatibility with Qiagen QIAcuity dPCR instruments was assessed using both 96-well and 24-well nanoplate formats. The 24-well plate format provides 26k partitions per well allowing for increased assay sensitivity, but lower sample throughout and higher cost per sample. Improved sensitivity provided by the 24-well nanoplate format was determined to be the best option for wastewater genotyping where the introduction of new variants can present at low circulating concentration within wastewater samples. Moreover, we determined that a minimum of five positive partitions is required for a reliable determination of variant fraction in the wastewater sample.

## Creating the Wastewater Processing and Genotyping SOP (Revision date April 03, 2023)

Successful implementation of the SARS-CoV-2 genotyping assay in combination with Nanotrap Particle wastewater sample processing was completed at Emory University using samples collected within the Atlanta metro region. The samples utilized for the verification study contained BQ.1, XBB or a combination of the two SARS-CoV-2 variants representing a transition period between BQ.1 and XBB. The Standard Operating Procedure (SOP) was developed based on the criteria established during this phase for both standard qPCR and dPCR methods (Figure S6). This SOP was implemented for Panel 1 of this study and is available upon request.


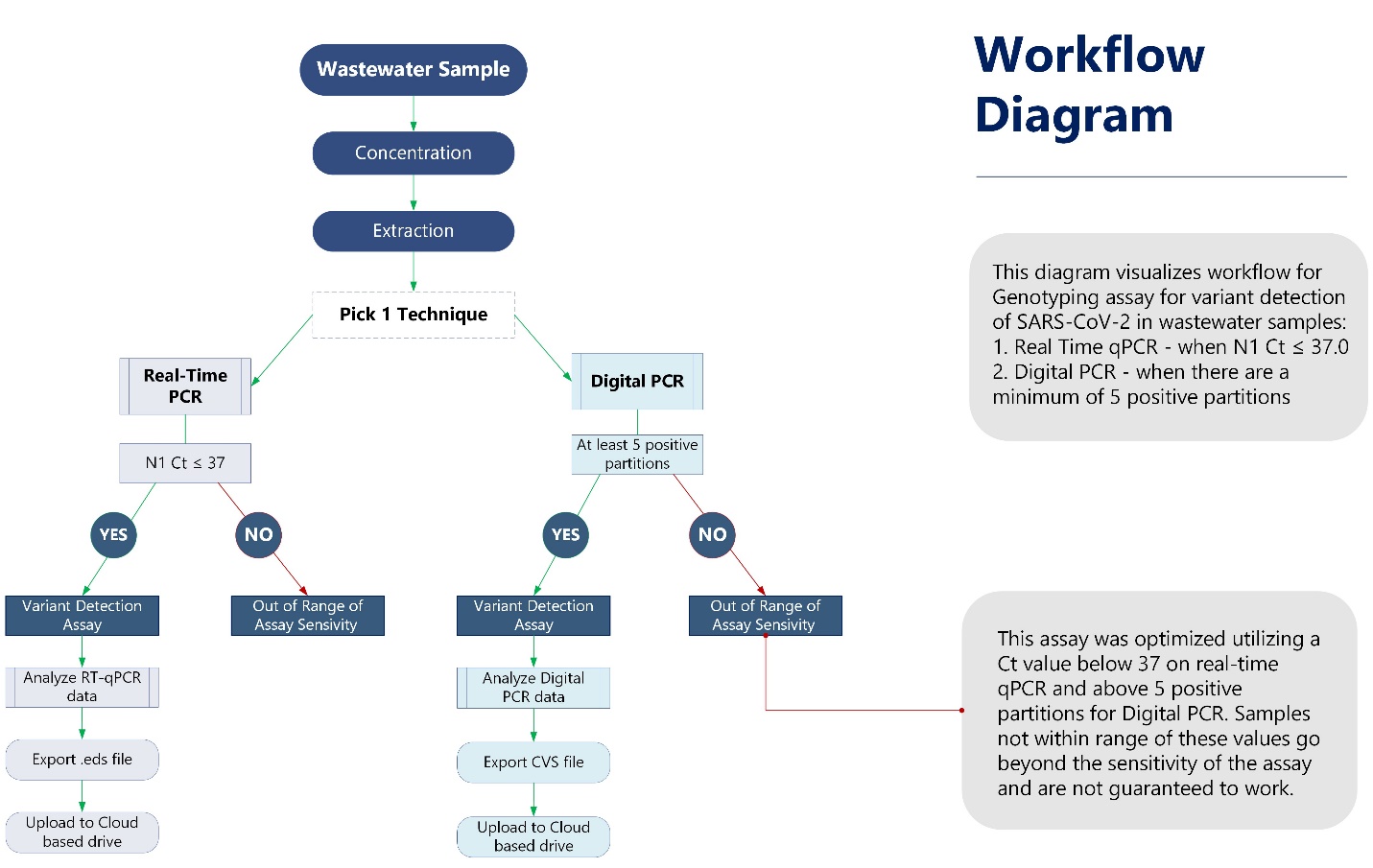


**Figure S6: Wastewater Genotyping Project Workflow Diagram:** This diagram outlines the genotyping assay process for detecting SARS-CoV-2 variants in wastewater samples, using either Real-Time PCR (N1 Ct ≤ 37) or Digital PCR (≥ 5 positive partitions). Successful assays proceed to variant detection, data analysis, and uploading results to a cloud-based drive.

Several quality control measures were implemented throughout sample processing, data analysis, and dashboard visualization. Each batch of wastewater samples included a Negative Extraction Control (NEC) to monitor for contamination during handling and extraction. For every dPCR run, two positive controls were included per variant assay: CTRL-0 (100% wild type, 0% mutant) and CTRL-100 (0% wild type, 100% mutant). These controls were designed in silico by ROSALIND Bio and synthesized using gBlocks™ (IDT) or GeneArt™ (ThermoFisher Scientific™).

Additionally, a No Template Control (NTC)—composed of the complete reaction mix with molecular-grade water in place of RNA—was included on each dPCR plate to detect potential reagent contamination. Positive controls were considered valid if they yielded at least five positive partitions. NEC/NTC controls were accepted only if fewer than five positive partitions were detected. Each run was reviewed by both the lab analyst and the ROSALIND platform, which automatically verified control presence (by name) and performance. Runs with failed controls were excluded from the dashboard. For dPCR variant calling, a minimum of five positive partitions per reaction well was required to classify a sample as positive for a specific variant.

All participating laboratories received standardized training on sample processing, data analysis, and uploading dPCR CSV files. To display data on the ROSALIND Wastewater Tracker dashboard, both PCR data and a complete metadata file were required. While metadata did not contain test results, it included critical details such as sample collection date and location (Table S6). The system automatically flagged samples with missing or mismatched metadata, and these were withheld from the dashboard until corrected by designated IT personnel at each site. Table S6 outlines the required metadata fields and their specifications.

**Table S6: Metadata requirements for ROSALIND Wastewater Tracker**

| **Field Name** | **Required** | **Comments** |
| --- | --- | --- |
| Lab_Name | Yes | For example “Emory”, … |
| Sample_ID | Yes | Need to be unique |
| Sample_Location | Yes | Name for the location of the collection of the sample |
| Sewershed_ID | Yes | Plant ID where the collection is connected |
| Sample_GPS | Yes | GPS coordinates for the location the sample was taken from |
| Sample_Matrix | Yes | See NWSS-Data-Dictionary_v3_1_5_20221122.xlsx (e.g.: primary sludge) |
| Sample_Type | Yes | See NWSS-Data-Dictionary_v3_1_5_20221122.xlsx (e.g.: grab) |
| State | Yes | We follow the ISO 3166-2 standard (<https://en.wikipedia.org/wiki/ISO_3166-2:US>) to have the US states information so, for example, California will be encoded as “US-CA” |
| County_Name | Yes | Official County name  <https://public.opendatasoft.com/explore/dataset/georef-united-states-of-america-county/table/?disjunctive.ste_code&disjunctive.ste_name&disjunctive.coty_code&disjunctive.coty_name&sort=year> |
| County_Code | Yes | Official County code  <https://public.opendatasoft.com/explore/dataset/georef-united-states-of-america-county/table/?disjunctive.ste_code&disjunctive.ste_name&disjunctive.coty_code&disjunctive.coty_name&sort=year> |
| Zip_Code | Yes | Zip code of the sample location |
| Population_Served | Yes | Number of people connected to the sample location |
| Collection_Date | Yes | In YYYY-MM-DD format (<https://en.wikipedia.org/wiki/ISO_8601#Calendar_dates>) |
| Genotyping_Date | No | In YYYY-MM-DD format |
| SARS-CoV-2_Concentration | No | SARS-CoV-2 concentration in the wastewater sample |
| Concentration_Unit | No | Unit used to report the SARS-CoV-2 concentration |
| Sequencing_ID | No | ID or URL to find the eventual sequencing results associated with this sample |
| Sequencing_Date | No | In YYYY-MM-DD format |

## Developing Genotyping Panel 1 (BQ.1 and XBB variants)

Following SOP Revision 1 (dated 04/03/2023), Emory University processed 86 wastewater samples collected from multiple sites in Georgia between April 11 and June 19, 2023. The assays were performed using the QIAcuity dPCR system, and the data were uploaded to the ROSALIND Tracker. As shown in Figure S7, the data reveal that the XBB variant was the most prevalent during this period (88.23%), followed by the BQ variant (9.03%), and an Unknown variant (2.73%). These results are consistent with clinical sample genotyping data from the same time frame, which showed 96.39% XBB, 2.41% BQ, and 1.20% Unknown. However, it is important to note the significant difference in sample coverage: only 83 clinical samples—representing individual patients—were genotyped, compared to the 86 wastewater samples that represent hundreds of thousands of individuals.

Although the marker sets used for wastewater and clinical samples are not identical, both datasets consistently indicate that XBB* and BQ* were the predominant SARS-CoV-2 variants circulating in Georgia during this time. This underscores the utility of wastewater-based surveillance in capturing variant prevalence at a community-wide scale, even when clinical sample availability is limited. (<https://tracker.rosalind.bio/tracker/dashboard/>).


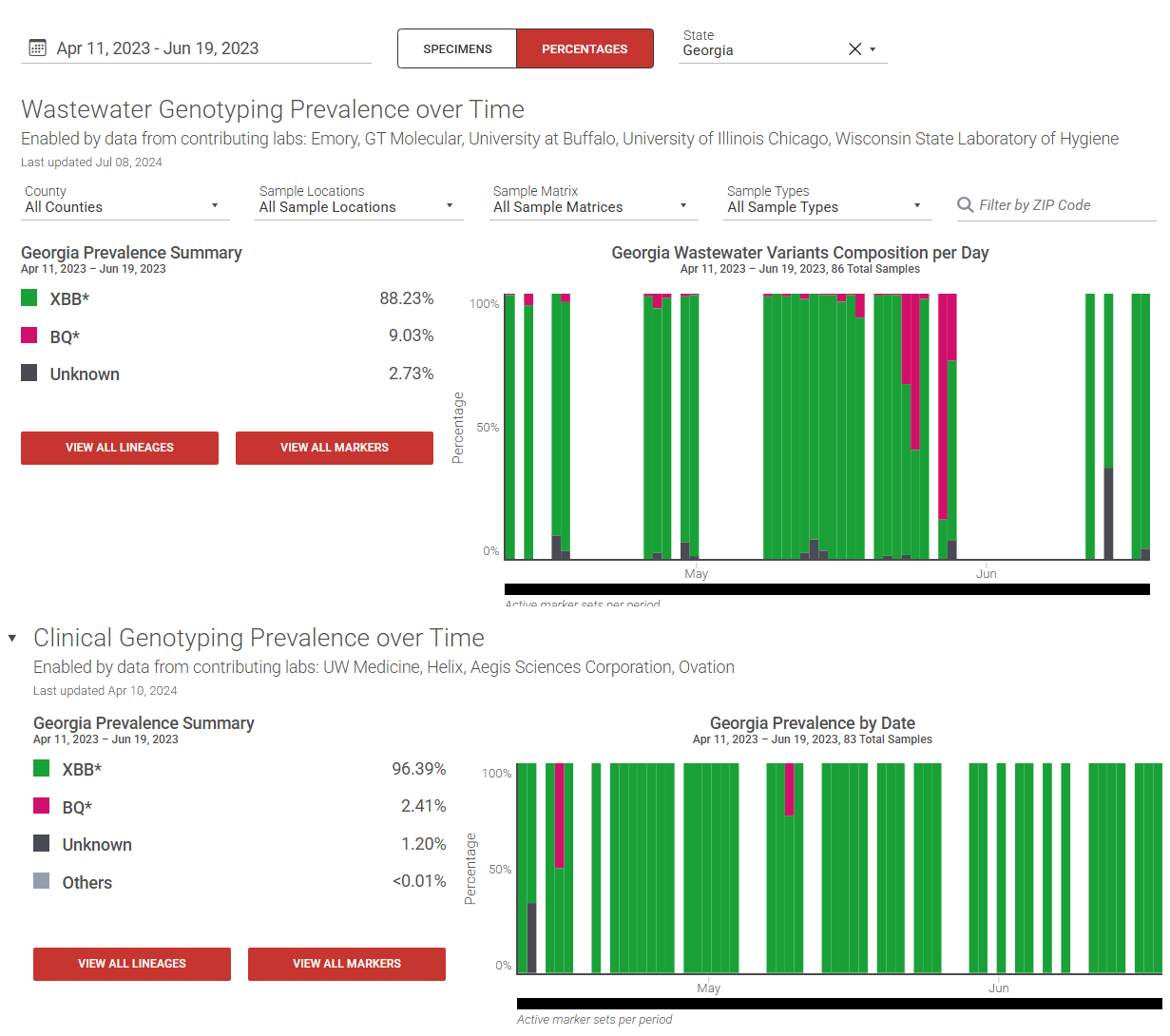


**Figure S7: Data from ROSALIND TRACKER, showing variant data from wastewater and clinical samples from the State of Georgia from April 11 to June 19, 2023.**

## Developing Genotyping Panel 2 (XBB, EG.1, EG.5, and FL variants)

At the end of June 2024, the NIH Variant Task Force determined that it was necessary to update the clinical sample and wastewater sample variant genotyping panel to monitor the following variants XBB, EG, FD, and FL. We hoped to use the same assays for wastewater genotyping as were being used for clinical sample genotyping. Because the XBB and BQ assays had worked well for wastewater-based dPCR genotyping, we expected that the new EG, FD, and FL assays would also work. This was true for the EG assay, which provided results for wastewater samples on the dPCR system using the same assay conditions as XBB and BQ. Unfortunately, the FD and FL assays did not work on the dPCR system for wastewater samples under these conditions.

We noticed two problems with the FD assay. It was experiencing double amplification on the dPCR and the wild type control for FD was amplifying. ROSALIND reported that the clinical labs testing the FD assay were also experiencing similar challenges with it. Ultimately, this assay was discarded and not implemented at the panel level.

The FL assay, when run under the dPCR conditions that were optimized for XBB and EG, had very little separation between the negative and positive partitions. Emory, ROSALIND, and Ceres evaluated multiple solutions to this, including longer probe lengths, manual threshold setting, and altering the dPCR assay parameters. The longer probes did not resolve the issue, and manual threshold setting on a plate-by-plate basis was deemed too onerous as a resolution, so we ultimately determined that the best approach assay was to alter the dPCR conditions. The key changes that were made were lengthening the Denaturation and Annealing / Extension times and lowering the Annealing / Extension Temperature. See Figure S8 for an example of how changing the assay conditions improved the results on the dPCR system for this assay.


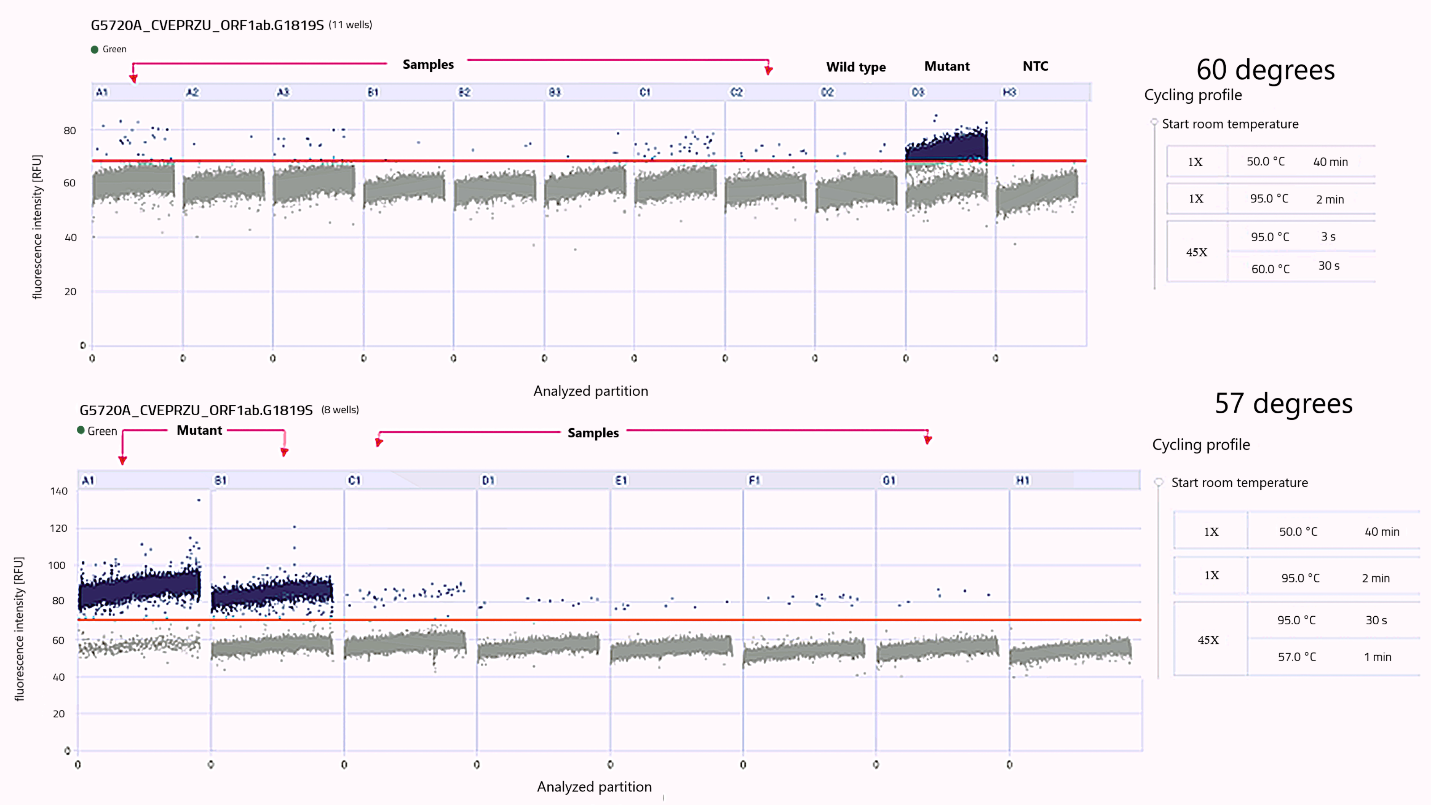


**Figure S8: FL assay performance on the QIAcuity dPCR system was dramatically improved when the denaturation and annealing/extension conditions were slightly altered**. The top frame is the PCR thermocycling conditions used for XBB and EG assays. The bottom frame shows updated conditions that were selected for the FL assay. Partitioning improved by increasing the denaturation & annealing/extension time and decreasing the annealing/extension temperature.

By the end of September, we had successfully verified that XBB, EG1, and FL (with modified dPCR conditions for FL) could be used in a panel for wastewater samples.

In mid-September 2024, the NIH Variant Task Force again decided to update the variant genotyping panel to monitor the following variants XBB, EG.1, EG.5, and FL. We started by running a validation process for the 0%, 50%, and 100% control samples across all four of these assays using the qPCR QuantStudio platform. These assays performed well in this context. In October, we then ran 73 archived wastewater samples collected from sites in Georgia between June 13 and September 2, 2023 using these assays. The results confirmed that these assays worked for wastewater samples. As shown in Supplementary Figure S9, the data indicate that the XBB variant was the most prevalent in Georgia during this period, with consistent findings between wastewater and clinical samples (72.81% in wastewater samples and 66.47% in clinical samples). The XBB.1.9 (FL/EG.5) variant was the second most prevalent, accounting for 22.71% in wastewater samples and 25.75% of clinical samples (<https://tracker.rosalind.bio/tracker/dashboard>).

We also noted that despite the fact that there were 2 times fewer wastewater samples than clinical samples tested in Georgia during this time period, that the FL and EG.5 variants were detected 22 days and 31 days earlier in the wastewater samples than in the clinical samples (Figure S9).


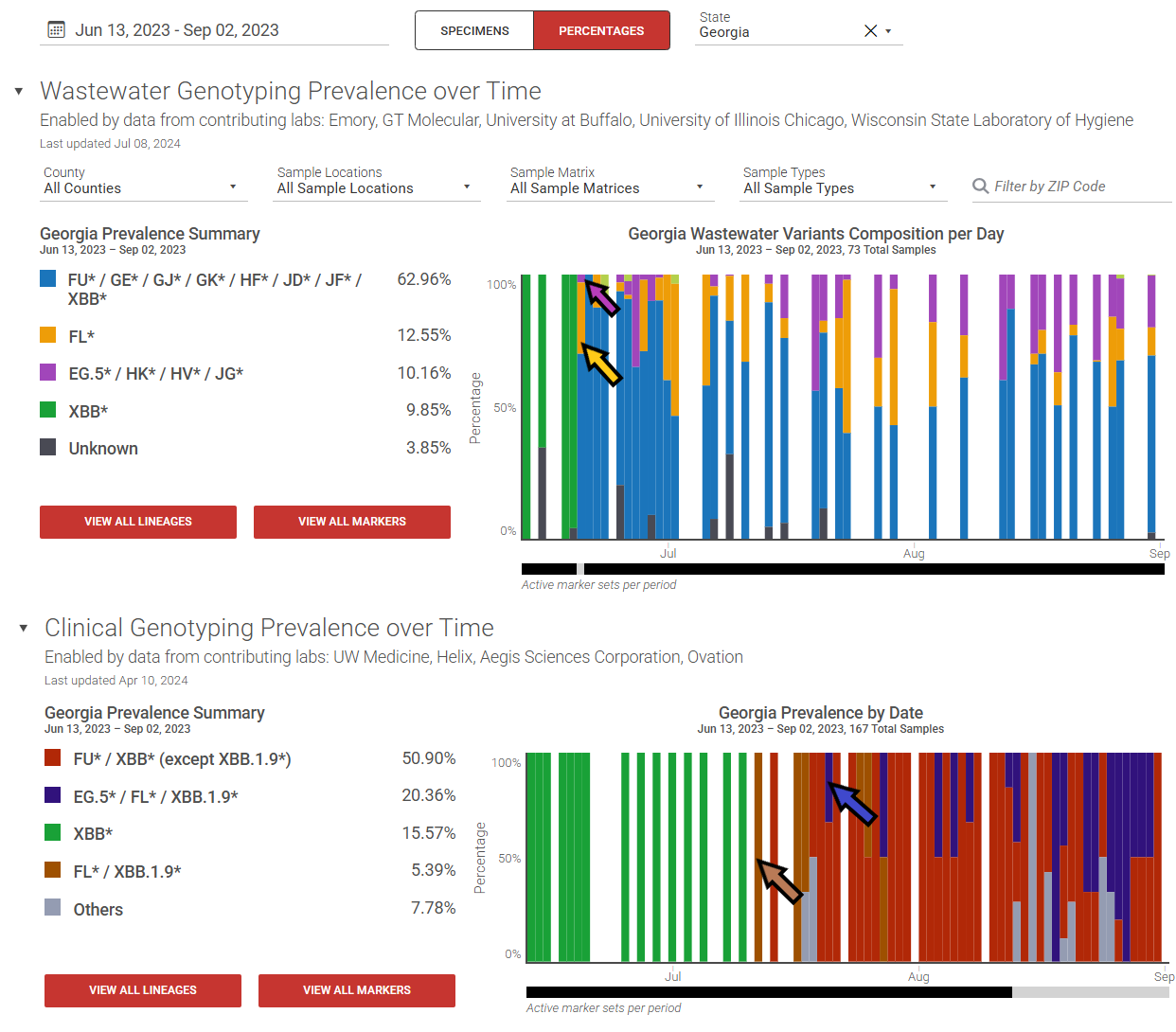


**Figure S9: Data from ROSALIND TRACKER, showing variant data from wastewater and clinical samples from the State of Georgia from June 13 to September 2, 2023.** June 20, 2023 first detection of FL and EG.5 variants in wastewater samples (Yellow and purple arrows). July 12, 2023 first detection of FL variant in clinical samples (brown arrow). July 21, 2023 first detection of EG.5/FL variant in clinical samples (navy blue arrow).

At this point, the SOP was updated to include the details on the new variant panel and assay settings to be used in the expanded pilot phase of this project. This new version was named “Variant detection SOP-Rev.1-1” and is dated 11/14/2023. The SOP is available on Ceres Nanosciences’ website at the following URL: <https://www.ceresnano.com/post/study-protocol-for-sars-cov-2-variant-detection-in-wastewater>.

## Developing Genotyping Panel 3 (XBB, EG.5, FL, JN)

Shortly after the five wastewater testing laboratories began the wastewater genotyping using the assays for Panel 2 (XBB, EG.1, EG.5, and FL), a new variant (JN) started to rapidly increase in prevalence in the United States. During early 2024, we evaluated two assays for the markers ANDKJKV and ANCFPZX. The ANDKJKV assay, under the dPCR conditions used for XBB, EG.1, and EG.5), exhibited double bands in both WT and MUT channels and false positive partitions (<5) in the WT channel for CTRL-100 (mutant homogeneous control). We evaluated longer PCR assay conditions, which resolved the false positive issue (Figure S10).


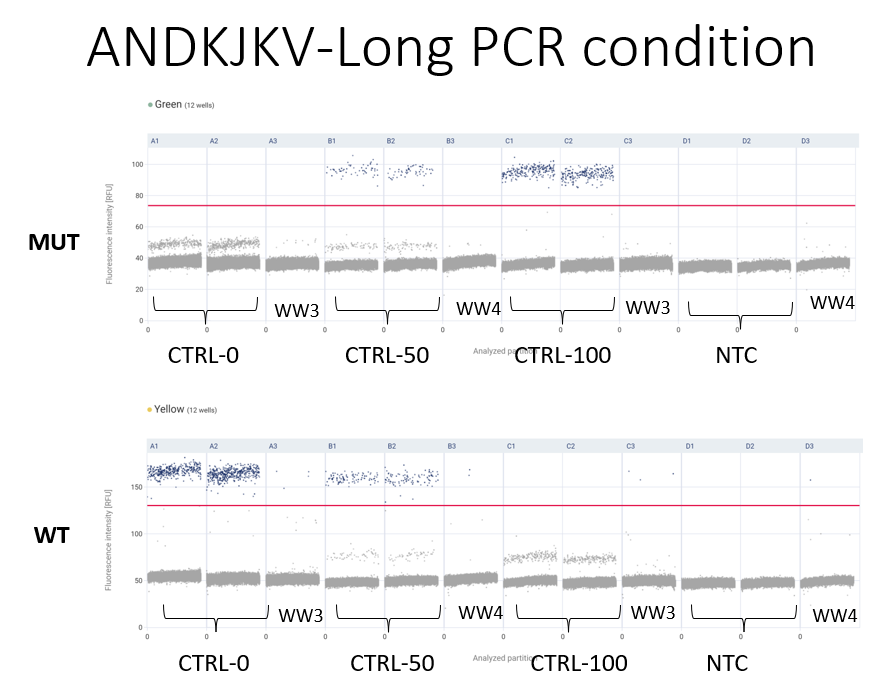


**Figure S10: The performance of the JN variant genotyping assay ANDKJKV improved under the longer PCR conditions established for the FL assay in Panel 2.** The top graph shows the Mutation Channel, and the bottom graph displays the Wild Type Channel. Double bands were observed in both channels, which can be accurately distinguished by proper thresholding to identify true positive partitions. Controls and samples performed as expected under these conditions. CTRL-0 represents 0% mutation, CTRL-50 represents 50% mutation, and CTRL-100 represents 100% mutation. NTC indicates the No Template Control, and WW represents wastewater samples.

Similarly, the ANCFPZX assay showed double bands in WT and MUT channels, but the right threshold could distinguish between false and true positive partitions. Sporadic false positives (<5 partitions) were observed in CTRL-0 (wild type homozygous control) in the MUT channel. Longer PCR conditions brought the double bands closer together, complicating thresholding. Considering performance and higher result quantity, as well as shorter run time, ANCFPZX was selected for JN variant genotyping (Figure S11).


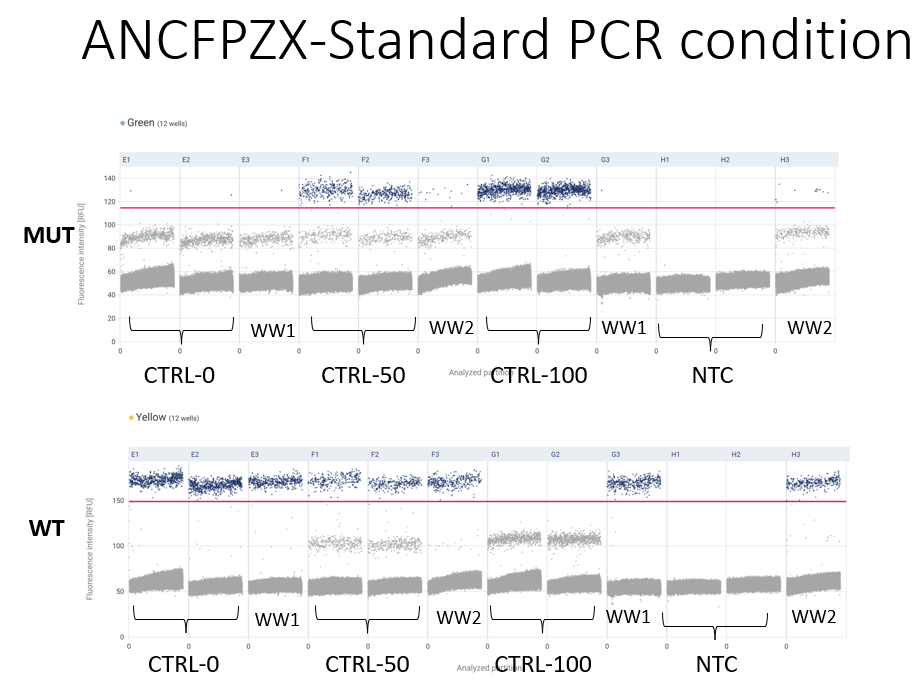


**Figure S11: The performance of the JN variant genotyping assay ANCFPZX under the standard PCR conditions established in Panel 2.** The top graph shows the Mutation Channel, and the bottom graph displays the Wild Type Channel. Double bands were observed in both channels, which can be accurately distinguished by proper thresholding to identify true positive partitions. Fewer than five false positive partitions were observed in CTRL-0 in the Mutation Channel. Other controls and samples performed as expected in both channels. CTRL-0 represents 0% mutation, CTRL-50 represents 50% mutation, and CTRL-100 represents 100% mutation. NTC indicates the No Template Control, and WW represents wastewater samples.

Validation of JN assay was completed by the end of January 2024. At this point, the SOP was updated to retire EG.1 assay and include the details on the new JN variant assay. This new version was named “Variant detection SOP-Rev.2” and is dated 02/02/2024. Reagent and supplies required for the new panel 3 were shipped to all five labs and instructed to continue wastewater testing using the new panel. Because JN had risen in prevalence more quickly than we could develop and validate the assay, we missed the rise of JN1 in real time (the ROSALIND Tracker dashboard showed a lot of unknown variants in January and February). So, we asked the labs to go back and retest any leftover RNA from January and February using the JN assay. These data were uploaded to the ROSALIND Tracker and were used to retrospectively update the dashboard.

## Georgia Wastewater Genotyping

Data from wastewater samples in Georgia was collected in January 2023 as part of the feasibility study for the wastewater genotyping project. Figure S12 presents genotyping results of wastewater samples in Georgia from April 11, 2023 to April 5, 2024 (52 weeks), highlighting the percentage of total markers in samples for different groups over a two-week period and the number of samples collected. Initially, the XBB* (purple line) marker dominated with nearly 100% presence in April 2023 but declined steadily to near 0% by December 2023. In contrast, the BA.2.86*/JN* (blue line) marker dramatically increased starting in January 2024, becoming the dominant variant by early 2024. The EG.5* (red line) marker shows fluctuations with a significant presence starting in July 2023, peaking at 50% in November 2023, and gradually declining. The BQ* (pink line) marker exhibits a sharp spike and decline in May 2023, disappearing by June. The FL* (cyan line) marker fluctuated throughout the year, peaking in August 2023, and maintained a consistent low presence until the end of the period. The EG1* (orange line) marker had a brief spike around December 2023 but otherwise maintained a low presence. The black dotted line represents the number of samples collected, averaging 20 samples bi-weekly and remaining steady for a total of 528 wastewater samples during this period.


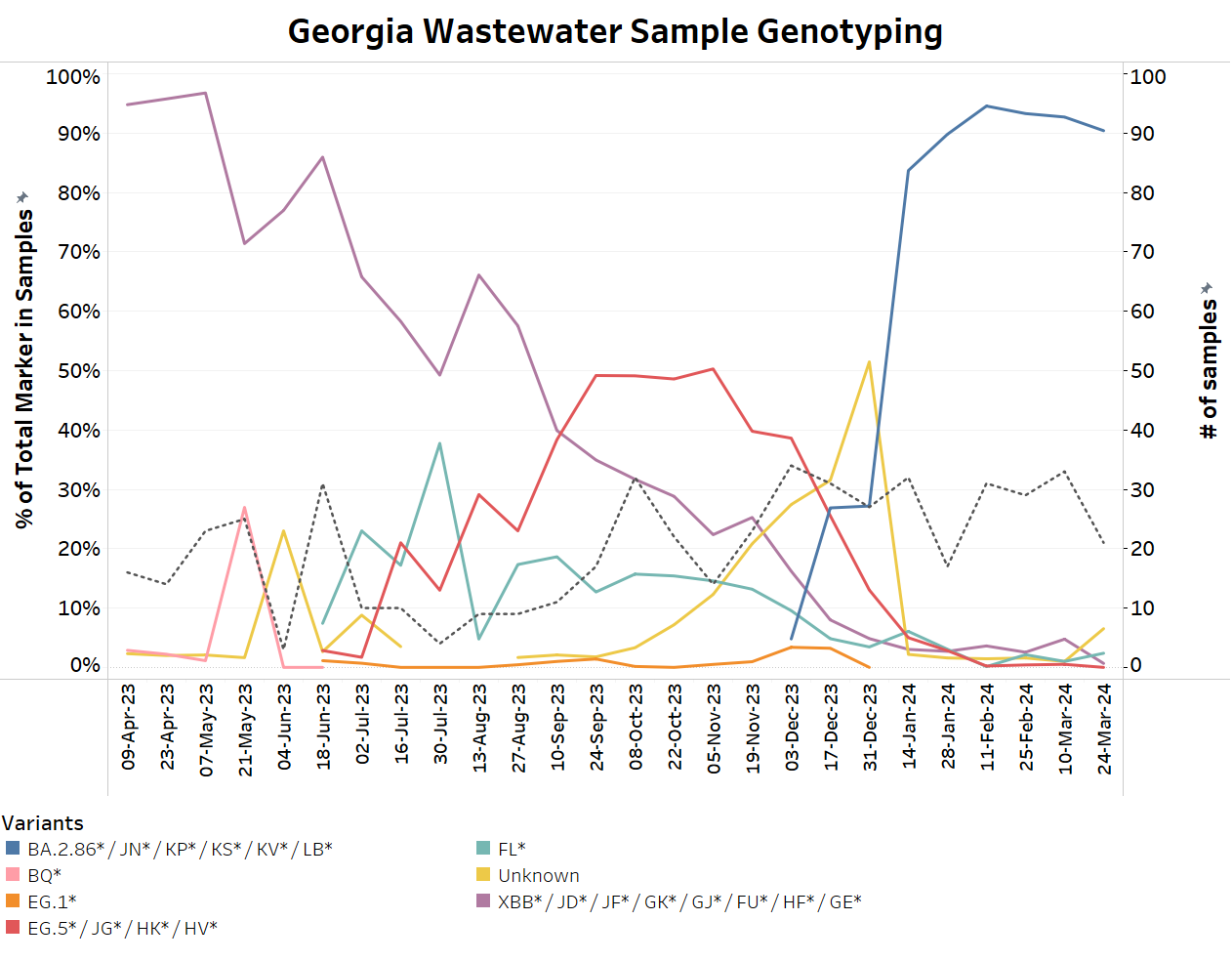
**Figure S12: Temporal Distribution of SARS-CoV-2 Genotypes in Georgia Wastewater Samples (April 2023 - April 2024)**. The graph shows the prevalence of various SARS-CoV-2 genotypes over time. Initially, XBB* (purple) dominated but declined by December 2023. BA.2.86* / JN* (blue) surged to 90% around February 2024. EG.5* (red) peaked at 50% in October 2023. FL* (cyan) showed sporadic peaks, while BQ* (pink) briefly peaked in May 2023. The Unknown marker (yellow) demonstrates an upward trend from October 2023 to January 2024, indicating the introduction of a new variant in the wastewater. The black dotted line represents the number of samples collected, staying steady around 20 samples per two weeks. The data indicate shifts in viral genotypes, suggesting changes in infection patterns or new variant introductions.

The Unknown (yellow line) marker shows periodic spikes, particularly in June and November 2023. Interestingly, the upward trend from October 2023, rising to about 50% in January 2024, experienced a sharp decline after transitioning to the new panel with the JN assay in February 2024. This likely indicates that the unknown marker was indeed the JN variant, and its rising prevalence was a precursor to the later dominance of the BA.2.86*/JN* variant. Retrospective analysis of a small set of samples collected between December 2023 and January 2024 confirmed the Unknown variant as JN variants (Figure S13). This underscores the importance of continuous assay development and implementation in accurately identifying and tracking emerging variants. We suggest studying the trend and setting a threshold of Unknown variant detection in wastewater samples to trigger new assay design and required validation works. This ability to rapidly identify new variants is crucial for timely public health responses and interventions. The emergence and identification of the JN variant highlight the dynamic nature of SARS-CoV-2 evolution and the ongoing need for adaptive surveillance strategies.

The graph depicting the genotyping results from wastewater samples in Georgia from April 2023 to April 2024 reveals three distinct phases of SARS-CoV-2 variant dynamics. The initial phase, from April to August 2023, is marked by the high prevalence of the XBB* variant, which starts near 100% and gradually declines, indicating a transition away from its dominance. The second phase, spanning August 2023 to December 2024, highlights the emergence and fluctuation of several new variants, including EG.5* and FL*. During this period, the unknown marker, later identified as the JN variant, begins to rise in prevalence from October 2023, underscoring the importance of adaptive assay development for accurate variant identification. The final phase, from December to April 2024, is characterized by the rapid rise and dominance of the BA.2.86*/JN* variant, which becomes the predominant variant by February 2024. This period also marks the replacement of the previously unknown marker by the identified JN variant, reflecting its significant circulation since October 2023. These observations underscore the dynamic nature of SARS-CoV-2 variants and the critical need for continuous monitoring and adaptive public health strategies to manage the evolving pandemic landscape.

The Unknown/BA.2.86*/JN* group's rapid rise starting in October 2023 suggests a significant event, such as an outbreak or increased transmissibility, making it the dominant variant by February 2024. The BQ* group's sharp spike in May 2023 and disappearance by June suggest a brief, unsustained outbreak. The EG.5* group's fluctuations and peak in November 2023 indicate periodic surges. While the FL* group's fluctuations suggest localized outbreaks or transient competitive advantages, the consistently low prevalence of EG.1 indicates it did not achieve widespread transmission.

## JN vs. Unknown variant

Figure S13 compares the prevalence of the BA.2.86*/JN* variant and the Unknown variant in 23 wastewater samples collected between December 11, 2023, and January 1, 2024. Initially analyzed using Panel 2 (including EG.5, EG.1, FL, XBB), these samples were later re-analyzed with a JN-specific assay to determine the prevalence of the JN variant. The graph reveals a high degree of correlation between the Unknown variant detected in the original Panel 2 results and the BA.2.86*/JN* variant identified using the JN-specific assay, suggesting that the Unknown variant is likely the BA.2.86*/JN* variant. Both variants show similar trends and fluctuations, with prevalence ranging from 40% to 80% and notable spikes around December 17, December 23, and December 28, 2023. This consistency confirms that the Unknown marker detected in the initial analysis corresponds to the BA.2.86*/JN* variant.


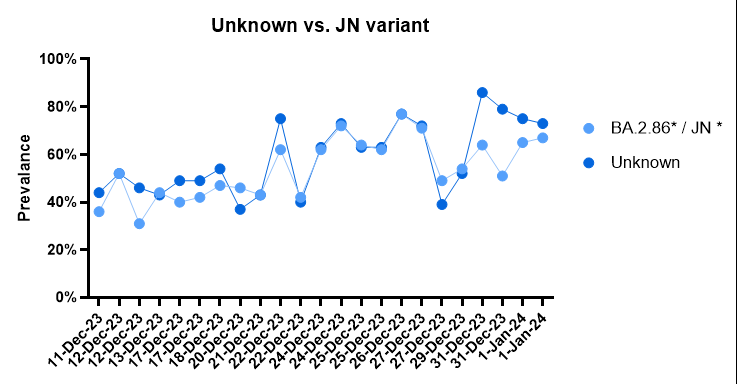


**Figure S13: Comparison of Unknown and JN Variant Prevalence in Wastewater Samples (December 11, 2023 - January 1, 2024).** The high correlation between the Unknown variant and the BA.2.86*/JN* variant suggests that the Unknown marker from the original Panel 2 analysis is likely the BA.2.86*/JN* variant. Both variants show similar trends and fluctuations, with prevalence ranging from 40% to 80% and notable spikes in mid to late December 2023.

## Georgia Clinical Genotyping

The graph in Figure S14 presents genotyping results of SARS-CoV-2 from clinical samples collected from patients who visited clinics in Georgia from April 9, 2023 to February 8, 2024. Initially, the XBB* (purple line) variant dominated with nearly 100% prevalence in April 2023, but it showed a steady decline starting in June 2023, fluctuating at 20% by February 2024. In contrast, the BA.2.86*/JN*- Unknown (blue and Yellow line) variants dramatically increased starting at the end of October 2024, becoming the dominant variant by early 2024. As we discussed in the previous section, the upward trend of the Unknown marker from October 2023 to January 2024, indicating the introduction of a new variant. The Unknown variant was identified as the JN variant after assay introduction.

A small fraction of the patient population (<10%) had the BQ* (pink line) variant in April 2023 which disappears in May 2023. Two new variants (EG.5 and FL) were detected in July 2023. The EG.5+/JG+/HK2+/HV* (red line) variant peaking at about 86% of patients in November 2023 before gradually declining. The FL* (cyan line) variant fluctuated between July to November, but generally maintained 20% of patients. The clinical genotyping dashboard also had a panel for both EG5 and FL which is indicated by Brown color. Considering both EG5 and FL (Red, Cyan, Brown) markers, these two variants dominated the clinical genotyping from September 2023 to December 2023, with a sudden replacement with the Unknown (yellow) variant.

The average number of patient samples collected during this period (black dotted line) was 13 per bi-weekly period, totaling 330 samples. The number of patient samples for genotyping testing sharply dropped from 62 samples in the first half of August 2023 to 2 samples at the end of September 2023. Eventually, no clinical samples were tested after February 8, 2024. Many factors might impact the number of clinical samples, including changes in population immunity, virus transmission rate, healthcare seeking behavior, and policy changes.

It is also worth noting that the low number of clinical samples caused gaps and unsmoothed line graphs, making interpretation challenging, particularly from September 2023 to the end of the study period. However, the clinical sequencing data from GISAID can be used to validate the results of the clinical genotyping dataset since genomic surveillance continued after the expiration of the COVID-19 public emergency declaration to help identify and monitor SARS-CoV-2 variants (7).


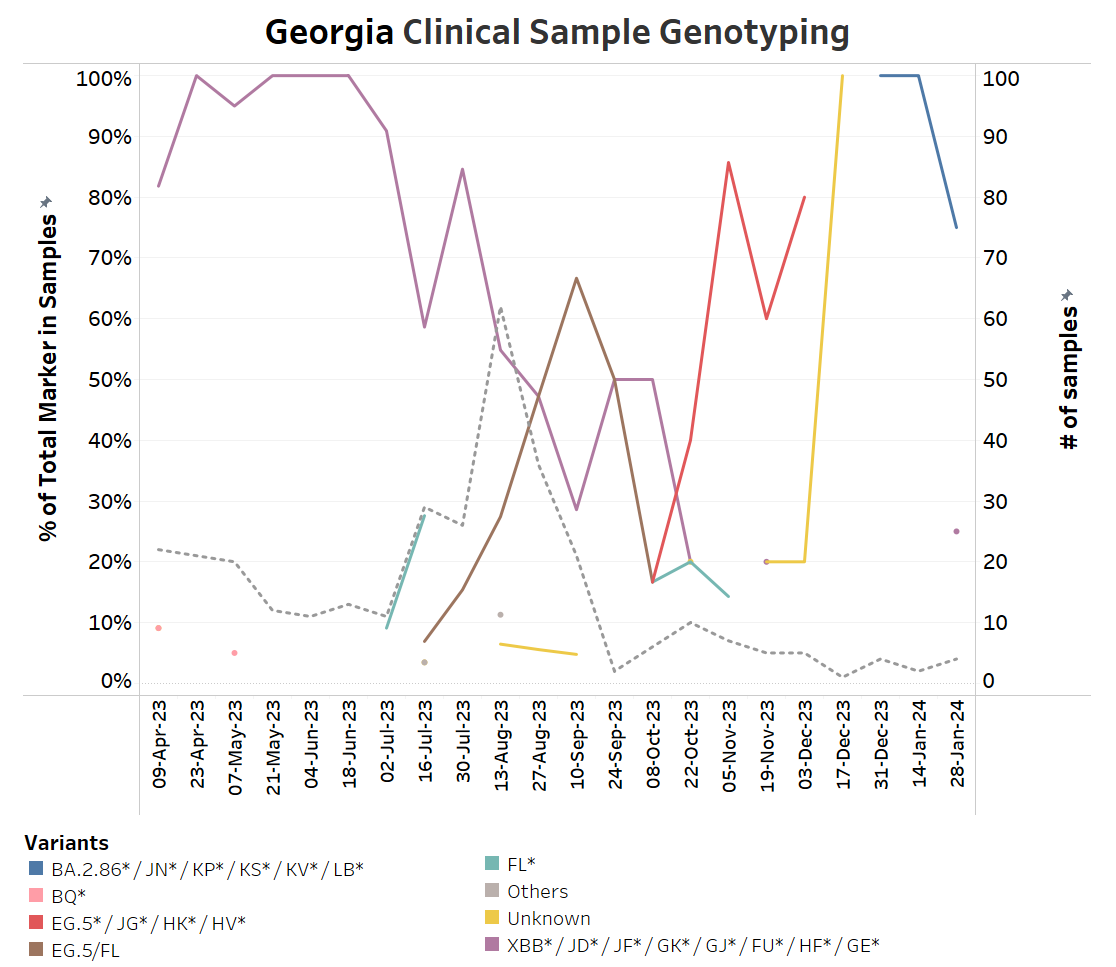


**Figure S14: Temporal Distribution of SARS-CoV-2 Genotypes in Georgia Clinical Samples (April 2023 - February 2024).** The graph presents the genotyping results of clinical samples in Georgia from April 2023 to February 2024, showing the prevalence of various SARS-CoV-2 genotypes and the number of samples collected. Initially, the XBB* (purple) genotype dominated, nearly 100% in April 2023, but declined steadily to around 50% by October 2023 and further dropped towards the end of the period. The BQ* (pink) genotype had very low presence, briefly peaking in April- May 2023 before disappearing. The EG.5* and FL* (red, cyan, brown) variants appeared in July 2023 and dominated the patient samples from September to December 2023, prior to sudden replacement with an Unknown/ JN* variants. The dotted black line represents the number of samples which experienced a sharp decline at the end of August. No clinical samples have been tested after February 8, 2024.

The clinical genotyping data demonstrates similar phases as the wastewater genotyping data. The first phase, from April to August 2023, is marked by the initial dominance of the XBB* variant with nearly 100% prevalence, and the brief emergence of the BQ* variant, which spikes in April but disappears in May. The second phase, spanning August to December 2023, sees the rise and fluctuation of several new variants, most notably the EG.5+/JG+/HK2+/HV* variant, which peaks in November 2023, and the FL* variant, which shows periodic increases. During this period, the Unknown variant begins to display significant spikes, particularly in October. The third phase, from December to April 2024, is characterized by the dramatic rise of the BA.2.86*/JN* variant, becoming the dominant strain by February 2024. Concurrently, the Unknown variant is identified as the JN variant following the introduction of a specific assay, leading to a sharp increase and peak in February 2024. This genotyping data suggests dynamic shifts in viral genotypes, reflecting changes in infection patterns or the introduction of new variants in the population over the study period.

## Georgia GISAID Sequencing

Figure S15 presents sequencing results of SARS-CoV-2 variants in clinical samples from Georgia, accessed from the GISAID database. The data covers the period from April 9, 2023 to April 5, 2024, presented in two-week intervals. It shows the percentage of total samples for different variant groups over time and the number of samples collected. Initially, the XBB* variant (purple line) shows high prevalence, starting near 100% in April 2023, but steadily declines to around 2% by February 2024. The BA.2.86*/JN* variant (blue line) dramatically rises starting in November 2023, becoming dominant by early 2024 and peaking at nearly 100% by April 2024. The EG.5+/JG+/HK2+/HV* variant (red line) fluctuates, peaking in October 2023 before declining. The BQ* variant (pink line) and EG1* (Orange line) exhibit a low fraction of the clinical samples (<3%) between April to July 2023. The FL* variant (cyan line) maintains low and fluctuating presence throughout the period. The Others category (yellow line) exhibits periodic spikes, before declining towards the end of the period. In this period, 2855 samples were analyzed with an average of 110 samples per bi-weekly interval. The number of samples collected (black dotted line) peaks around August 2023 and January 2024, indicating increased sampling activity, but significantly declines at the end of the study period.


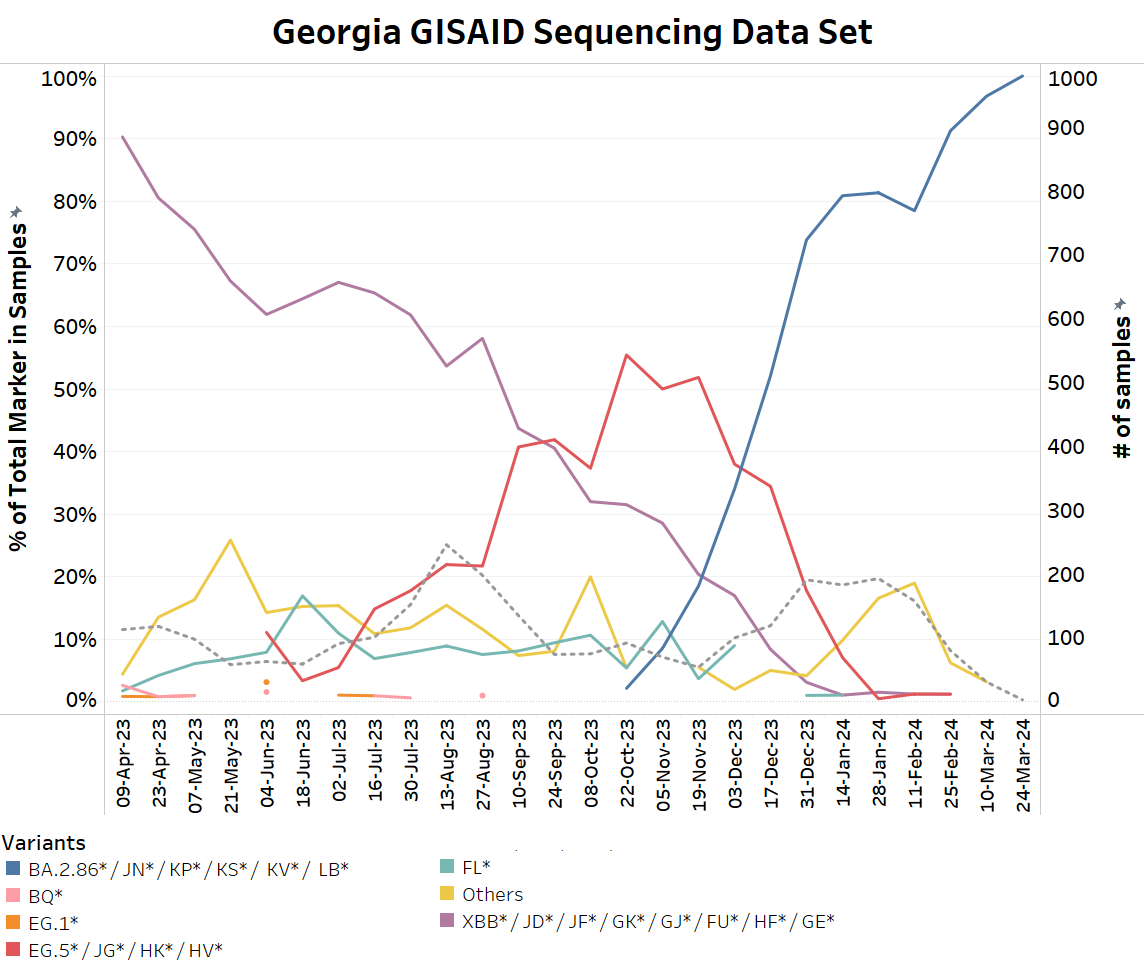


**Figure S15: Temporal Distribution of SARS-CoV-2 Genotypes in Georgia Clinical Samples from GISAID Database (April 2023 - April 2024).** The graph illustrates the genotyping results of SARS-CoV-2 from clinical samples in Georgia, as recorded in the GISAID database, spanning from April 2023 to April 2024. It shows the prevalence of various SARS-CoV-2 genotypes over time and the number of samples collected. XBB (purple): Initially dominant, comprising nearly 100% of samples in April 2023, but declined steadily to 0% by January 2024. BQ and EG1 (pink, orange): Had very low presence in April and July 2023 before disappearing entirely. FL (cyan): Showed consistently low prevalence from April to December 2023 and disappeared in January 2024. EG.5 (red): Emerged in June 2023, dominated patient samples in October and November 2023, and was suddenly replaced by JN variants. The dotted black line represents the number of samples, averaging 110 samples in bi-weekly increments. The number of samples significantly reduced in February 2024.

The initial dominance and subsequent decline of the XBB* variant, alongside the rapid rise and dominance of the BA.2.86*/JN* variant starting in November 2023, suggest significant shifts in the variant landscape due to factors like increased transmissibility or immune escape. The fluctuations of the EG.5+/JG+/HK2+/HV* variant and periodic spikes in the Others category indicate ongoing viral evolution and the emergence of various other variants. The brief presence of the BQ* variant underscores the potential for transient outbreaks. Increased sampling activity, particularly around August 2023 and January 2024, likely reflects heightened surveillance efforts in response to emerging variants, underscoring the importance of agile and responsive public health measures.

## dPCR genotyping vs. NGS sequencing for variant detection in wastewater: Cost-Time-Labor analysis

Due to the complexity and the time-intensive nature of library preparation and bioinformatic analysis protocols, sequencing is often more expensive and has a longer turnaround time than real-time qPCR (RT-qPCR) (8-10). We conducted a cost-time-labor analysis from the perspective of a typical wastewater surveillance lab that is starting with extracted nucleic acids in order to understand the difference in cost and turnaround time for the dPCR genotyping method used in this study compared to the whole-genome sequencing (WGS) amplicon-tiled sequencing approach on the Illumina platform by NGS technology. Our calculation is based on analyzing 17 wastewater samples at a time, which is the throughput of the wastewater genotyping method described here. The sequencing techniques used in this calculation are described in the method section under the Wisconsin State Laboratory of Hygiene sequencing method. Table S7 summarizes the findings.

The dPCR genotyping assay requires two positive controls for SNP mutation and the wild-type allele (CTRL-100, CTRL-0). Additionally, one Non-Template-Control (NTC) is required to control the PCR reagent. In comparison, sequencing requires one positive and one negative control. This increases the number of tests for dPCR to 21 and sequencing to 20 tests.

Using these assumptions, we estimate that each dPCR run costs $375. Since we used single-plex assays in this study, each panel required four plates, increasing the total cost to $1500 and $88 per wastewater sample. In contrast, sequencing requires only one run but at a higher cost of $2345 per run, which is $138 per wastewater sample. Considering the current single-plex setup, the cost of reagents for the dPCR genotyping assay is 36% lower than the standard sequencing workflow. A significant advantage of the single-plex method is the ability to quickly switch out individual markers without the need to revalidate an entire multiplex assay, providing flexibility and reducing validation efforts. However, with multiplexing, the cost per run can be reduced by up to 84%, bringing it down to approximately $22 per sample.

The dPCR workflow involves fewer steps compared to the multi-step sequencing workflow (which includes quality control, library preparation, and cleaning). A dPCR run usually takes between 2-3 hours. Data analysis and interpretation require thresholding and exploring the CSV file, which do not require extensive technical skills. The results provide the target variant quantity in the samples, which can be manually or automatically converted into genome copies per wastewater volume or prevalence in the wastewater. For the final protocol, we ran four separate dPCR assays to generate data on four variants of interest, with a total sample-to-result time of about 12 hours. By multiplexing the dPCR assay, the run time could be shortened to 4 hours. The single-plex method's flexibility in switching out markers quickly without extensive revalidation is particularly beneficial for adapting to emerging variants and rapidly updating surveillance protocols.

On the other hand, sequencing runs require significant computing time to generate reads, and the output needs bioinformatic tools to convert it into actionable data on variant prevalence in wastewater, typically done through an automated pipeline. The sequencing method requires a higher degree of expertise to generate valid and reliable results compared to the dPCR method. The sample-to-result turnaround time for the sequencing method is 38 hours. Despite using singleplex assays, the dPCR method used in this study is 68% faster. Adapting these dPCR assays to a multiplex format could potentially reduce the turnaround time by 90%, to less than a working day. This calculation does not consider limitations in sample shipment, logistics, and scheduling. In practice, a typical sequencing sample-to-result process might take weeks to be reported back to the client. In reality, a typical sequencing sample-to-result process might take weeks to be reported back to the customer.

**Table S7: Comparison of dPCR Genotyping Method and WGS Amplicon-Tiled Sequencing Approach.** This table compares the costs, sample processing requirements, and time to results for the dPCR genotyping method and the whole-genome sequencing (WGS) amplicon-tiled sequencing approach on the Illumina platform, based on 17 wastewater samples. The current singleplex dPCR method shows 36% lower reagent costs and a 68% shorter turnaround time compared to WGS. Multiplexing can further reduce reagent costs by up to 84% and the start-to-finish time by 90%.

|  |  | **dPCR** | **WGS** |
| --- | --- | --- | --- |
| Samples & Controls | Number of samples | 17 | 17 |
|  | Number of reagent blank | 1 | 1 |
|  | Number of controls | 3 | 2 |
|  | Number of Technical replicates | 1 | 1 |
|  | Number of assays | 1 | 1 |
|  | **Number of total reactions** | **21** | **20** |
|  |  |  |  |
| Reagent Cost | Sample prep cost (e.g. tips, maintenance, etc.) | $105 | $280 |
|  | QC reagent cost | $0 | $120 |
|  | Library prep reagent cost | $0 | $1020 |
|  | Sequencing/PCR per run cost | $270 | $920 |
|  | **Total cost per run** | **$375** | **$2,340** |
|  | Number of runs | 4 | 1 |
|  | **Total cost Per Panel** | **$1,500** | **$2,345** |
|  | **Cost per sample** | **$88** | **$138** |
|  |  |  |  |
| Sample to Result Time | Sample prep time (hour) | 1 | 4 |
|  | Instrument run/Computing time (hour) | 10 * | 30 |
|  | Analysis time (hour) | 1 | 4 |
|  | **Total start to finish time (hour)** | **12** | **38** |
|  | * For 4 plates |  |  |
|  |  |  |  |
| Instrument | Instrument | QIACuity Digital PCR system Four- 5plex | MiSeq, Biomek i-Series, QIAxcel, ABI QuantStudio 6 |
|  | Other requirement | NA | Linux Station + server (for storage) |
|  | instrument cost | $$ | $$$ |
|  |  |  |  |
| Labor | Number of Tech/Scientist | 2 | 2 |
|  | Level of expertise | Low | High |

## Wastewater Sampling Across Participated States

In total, 91 sites across six U.S. states—Georgia, California, Illinois, Louisiana, New York, and Wisconsin—participated in this study. Table S8 summarizes key details of the wastewater sampling protocols used at each site, including collection methods, frequency, sample volumes, and availability of environmental monitoring data.

**Table S8: Summary of Wastewater Sample Collection from Six States**

| **State** | **Collection site** | **Flow Type** | **Collection method** | **Sampling method** | **Frequency** | **Volume** | **Environmental monitoring data?** |
| --- | --- | --- | --- | --- | --- | --- | --- |
| California | 11 Correctional Facilities | Effluent | Automated | Time-Proportional 24-hour composite | Twice weekly | 40 mL | Not available |
| Georgia | 17 WWTPs | Influent | Automated | 24-hour time-proportional or flow-weighted composite | weekly | 250 mL | Temperature, pH |
| Illinois | 17 WWTPs + 1 WRD | Influent | Automated | 24-hour flow-proportional (n=9), time-proportional (n=6), volume-proportional (n=2), plus 1 grab sample | Twice weekly | 2x 50 ml | Arrival temperature, flow rate |
| Louisiana | 18 WWTPs | influent | Automated | Time-Proportional 24-hour composite | Twice weekly | 40 mL | Not available |
| New York | 7 WWTPs | Influent | Automated | Time- or flow-weighted 24-hour composite | Twice weekly | 500 mL | Temperature and weather not explicitly collected but available upon request from WWTPs |
| Wisconsin | 20 WWTPs | Influent | Automated | 24-hour flow-proportional; one site used time-weighted composite | Weekly | 250 mL | Arrival temperature, total flow, and optionally: in-stream temp, DO, conductivity, pH |

## Sample Processing Timelines and Storage Conditions

Across the six participating states, most wastewater samples were processed promptly upon arrival. When processing delays occurred, they were typically due to logistical factors such as sample shipping schedules or batching strategies for efficient lab workflow. In any delayed cases, wastewater samples were stored in short-term refrigerated condition (2–8°C) prior to nucleic acid extraction. Below is a summary of the processing workflows by state:

- **California**: Samples were processed immediately upon receipt. Occasional delays were due to unavoidable shipping issues.
- **Georgia**: Samples were stored at 4°C upon receipt and typically processed within 1–2 days, consistent with the weekly sampling schedule and outlined methods.
- **Illinois**: Upon arrival, all samples were stored at 4°C and processed every Thursday once all batch samples had been received.
- **Louisiana**: Samples were processed immediately upon receipt. Occasional delays were due to unavoidable shipping issues.
- **New York**: Samples were stored at 4°C and processed within 12 hours of arrival. Minimal delays were introduced to optimize lab throughput and resource use.
- **Wisconsin**: Samples were processed immediately. No notable delays occurred.

These practices reflect standard wastewater epidemiology protocols and ensure consistency and reliability across the testing network.

## Reference

1. Tamura T, Ito J, Uriu K, Zahradnik J, Kida I, Anraku Y, et al. 2023. Virological characteristics of the SARS-CoV-2 XBB variant derived from recombination of two Omicron subvariants. Nat Commun 14:2800. <https://doi.org/10.1038/s41467-023-38435-3>.
2. Kayikcioglu T, Amirzadegan J, Rand H, Tesfaldet B, Timme RE, Pettengill JB. 2023. Performance of methods for SARS-CoV-2 variant detection and abundance estimation within mixed population samples. PeerJ 11:e14596. <https://doi.org/10.7717/peerj.14596>.
3. Lai E, Kennedy EB, Lozach J, Hayashibara K, Davis-Turak J, Becker D, et al. 2022. A method for variant agnostic detection of SARS-CoV-2, rapid monitoring of circulating variants, and early detection of emergent variants such as Omicron. *J Clin Microbiol* 60. :e00342-22. <https://doi.org/10.1128/jcm.00342-22>.
4. Ma KC. 2023. Genomic surveillance for SARS-CoV-2 variants: circulation of Omicron lineages — United States, January 2022–May 2023. *MMWR Morb Mortal Wkly Rep* 72. <https://www.cdc.gov/mmwr/volumes/72/wr/mm7224a2.htm>.
5. Surie D. 2022. Effectiveness of monovalent mRNA vaccines against COVID-19–associated hospitalization among immunocompetent adults during BA.1/BA.2 and BA.4/BA.5 predominant periods of SARS-CoV-2 Omicron variant in the United States — IVY Network, 18 States, December 26, 2021–August 31, 2022. *MMWR Morb Mortal Wkly Rep* 71. <https://www.cdc.gov/mmwr/volumes/71/wr/mm7142a3.htm>.
6. Addetia A, Piccoli L, Case JB, Park YJ, Beltramello M, Guarino B, et al. 2023. Neutralization, effector function, and immune imprinting of Omicron variants. *Nature* 621:592–601. <https://doi.org/10.1038/s41586-023-06487-6>.
7. Silk BJ. 2023. COVID-19 surveillance after expiration of the public health emergency declaration ― United States, May 11, 2023. *MMWR Morb Mortal Wkly Rep* 72. <https://www.cdc.gov/mmwr/volumes/72/wr/mm7219e1.htm>.
8. Jayamohan H, Lambert CJ, Sant HJ, Jafek A, Patel D, Feng H, et al. 2021. SARS-CoV-2 pandemic: a review of molecular diagnostic tools including sample collection and commercial response with associated advantages and limitations. Anal Bioanal Chem 413:49–71. <https://doi.org/10.1007/s00216-020-02958-1>.
9. Ranasinghe D, Jayadas TTP, Jayathilaka D, Jeewandara C, Dissanayake O, Guruge D, et al. 2022. Comparison of different sequencing techniques for identification of SARS-CoV-2 variants of concern with multiplex real-time PCR. PLOS ONE 17:e0265220. <https://doi.org/10.1371/journal.pone.0265220>.
10. Tiwari A, Adhikari S, Zhang S, Solomon TB, Lipponen A, Islam MA, et al. 2023. Tracing COVID-19 trails in wastewater: a systematic review of SARS-CoV-2 surveillance with viral variants. Water 15:1018. <https://doi.org/10.3390/w15061018>.
